# Plant microbiome responses to bioinoculants and volatiles

**DOI:** 10.1186/s40793-025-00715-4

**Published:** 2025-05-21

**Authors:** Expedito Olimi, Martina Duller, Martina Stangl, Samuel Bickel, Angelika Battisti, Peter Kusstatscher, Wisnu Adi Wicaksono, Ahmed Abdelfattah, Tomislav Cernava, Gabriele Berg

**Affiliations:** 1 Institute of Environmental Biotechnology, Graz University of Technology, Graz, Austria; 2 School of Biological Sciences, Faculty of Environmental and Life Sciences, University of Southampton, Southampton, UK; 3 Leibniz Institute for Agricultural Engineering and Bioeconomy (ATB), Potsdam, Germany; 4 Institute for Biochemistry and Biology, University of Potsdam, Potsdam, Germany

**Keywords:** Plant microbiome, Sustainable agriculture, Volatile organic compounds, Bioinoculants, Microcosm experiments, Amplicon sequencing

## Abstract

**Background:**

There is an increase in the adoption of biological solutions for plant production as a means of attaining sustainable agriculture. A detailed understanding of the influence of specific bioinoculants and their volatile metabolites on native soil and plant microbiomes can improve future microbiome management practices.

**Results:**

Here, we examined the effect of bacterial inoculants and volatile compounds as individual and combined treatments on apple plant and soil microbiome. The study used specially designed microcosms that facilitated the separation of the different plant compartments. A compartment- and soil-specific effect of treatments on the native soil and plant microbiome was observed. The live bacterial inoculants as compared to their volatiles had a stronger effect on the plant and soil microbiome, particularly the root microbial community. The combined effect of bacterial inoculants was higher compared to volatiles (R^2^ = 5% vs. 3%). Treatment-specific effects were observed, like the influence of 2-butanone on the phyllosphere bacterial diversity, and an increase in fungal richness in Serratia-treated soils.

**Conclusions:**

Among the examined treatments, inoculation with bacteria compared to volatile metabolites induced more significant shifts within the plant and soil microbiome. This observation has implications regarding the merits of applying living microorganisms. The findings highlight the potential of microbiome management approaches for enhancing microbiota functions.

**Supplementary Information:**

The online version contains supplementary material available at 10.1186/s40793-025-00715-4.

## Background

Microbiome-based innovations support sustainable developments in agriculture by offering solutions to challenges such as climate change, declining biodiversity, and food insecurity [1]. The plant microbiota consists mainly of bacteria and fungi, which can stimulate plant immunity, suppress disease, supply nutrients, and protect the plant host from numerous biotic and abiotic stresses [2]. Thus, the deployment of microbiome-based solutions has enormous potential for ensuring sustainable plant production and stress resilience [1, 3]. The World Economic Forum (2018) recognized the immense potential of microbiome technologies in revolutionizing agroecosystems, mainly through reducing inorganic input in arable land [1]. Current intensive farming practices affect the microbiome of agroecosystems [4], and require special attention such as its management and restoration [5]. Numerous commercially available biological products have been applied in agricultural settings, yet there is limited knowledge regarding their impact on indigenous microbiomes.

Microbiome management includes applying (1) microbiome transplants, (2) microbes with beneficial properties (probiotics), (3) microbiota-derived compounds, and (4) changing environmental conditions in a way that shifts microbiomes to a healthy state [6, 7]. Traditional microbial transplants with soil organic amendments like manures [8] and biodynamic formulations [9] can help restore soil quality. Similarly, beneficial members of the microbiota that can enhance plant functioning, growth, and the suppression of pathogens [10, 11]. In addition, microbiota-derived metabolites like microbial volatile organic compounds (mVOCs) [12] provide an alternative tool for microbiome management. In general, mVOCs are characterized by low molecular weights and high vapour pressures at ambient temperature [13], and contribute to various ecological roles in the biosphere [14, 15]. Volatiles are essential as communication messengers and crop protectants through direct activity as well as the activation of plant immune responses [16]. In intercropping systems, they were found to play are role in controlling herbivore populations [17]. Moreover, certain mVOCs are used as biofumigants that can protect crops against phytopathogenic fungi, bacteria and nematodes [18, 19]. Despite a wealth of research on microbiome management in agroecosystems, translation of these strategies to commercial scales is rare but promising [20, 21]. However, before production is up to scale, a comprehensive assessment is needed to evaluate potential impacts of treatments on native microbiomes.

In this study, we focus on microbiome management approaches in agroecosystems that involve the application of bacterial inoculants and their associated volatile compounds. The applied bacterial inoculants include *Serratia plymuthica* HRO C48 and *Stenotrophomonas rhizophila* SPA P69 (henceforth referred as *Serratia* and *Stenotrophomonas*, respectively) [10, 22], which are known producers of bioactive volatile compounds [23]. For example, the volatile compounds that are emitted by *Serratia* showed antagonistic activity against soil-borne fungal pathogens [24]. In addition to this, it has proteolytic and chitinolytic activity [2], and can produce antibiotics like pyrrolnitrin [25]. *Stenotrophomonas*, just like *Serratia*, can suppresss fungal pathogens in the root of treated plants, and its antifungal effect can be partially attributed to volatile compounds [26, 27]. It also produces glucosyl glycerol and spermidine for amelioration of abiotic stress in plants [28]. While numerous studies have focused on deciphering the mechanisms underlying activity of microbial inoculants towards pathogens and with plant hosts, not many have examined the influence of bacterial inoculants and their volatile compounds on plant and native soil microbiomes.

Here, we examine (i) the impact of microbiome-based solutions that include plant-associated bacterial inoculants and volatile metabolites on native plant and soil microbiomes, (ii) the microbiome shifts and predicted functional diversity post-inoculation in two contrasting soils, (iii) the effect of bacterial treatments on the root ecosystem functions using carbon utilization assays. We hypothesized that bacterial inoculants have a stronger effect on plant and native soil microbiomes compared to their volatile compounds, because these compounds only partially account for the activity of bacterial inoculants. We used headspace solid phase microextraction with gas chromatography and mass spectrometry for the in vitro profiling of major compounds emitted by the bacterial inoculants. The bacterial inoculants and their associated volatiles were then inoculated in apple microcosms to observe plant and soil microbiome shifts using amplicon sequencing of 16S rRNA gene fragments and the ITS regions for bacterial and fungal community, respectively. In addition, we used Biolog Eco-plates to measure changes in carbon utilization patterns in the root.

## Methods

### Plant materials, treatment description and application

**Fig. 1:**
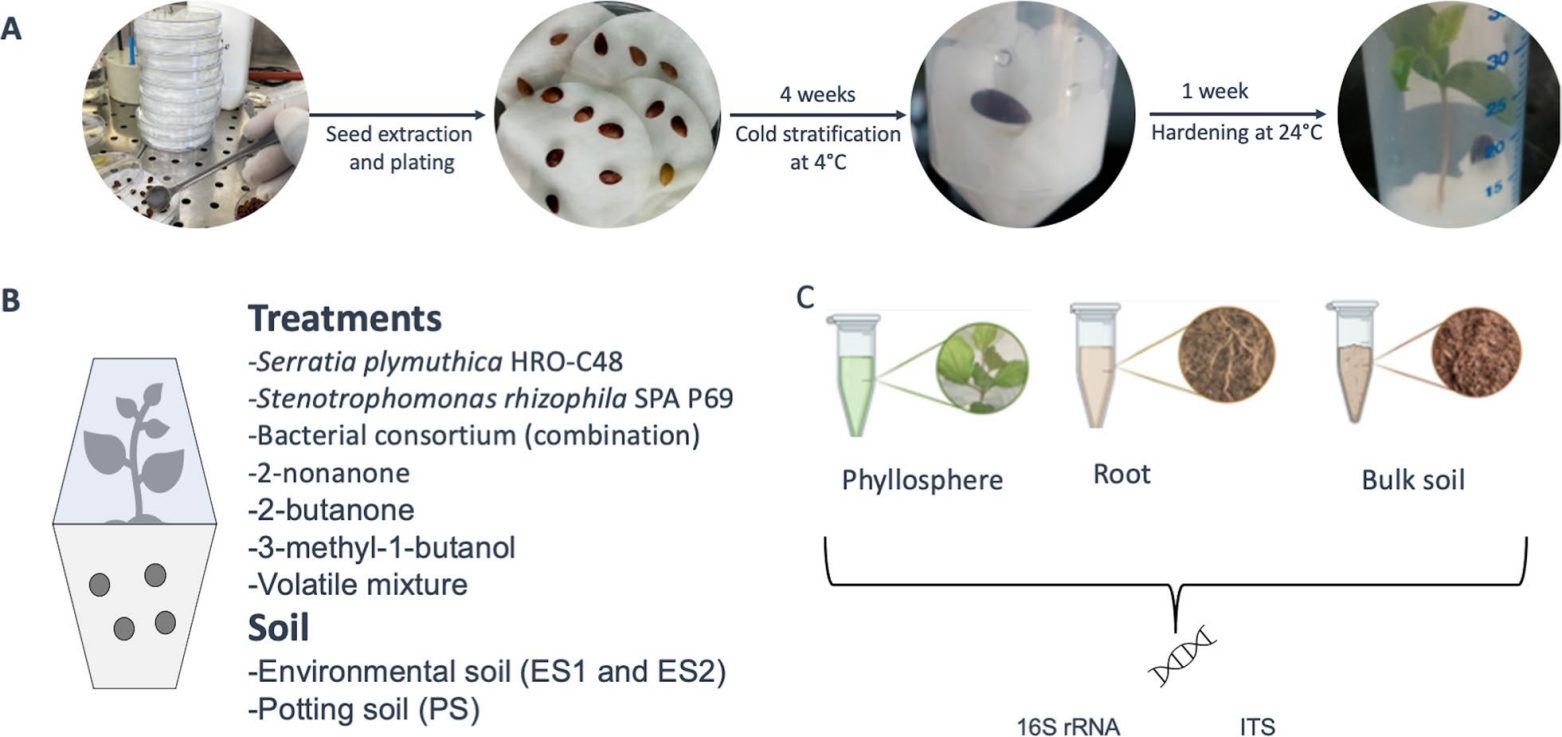
Schematic representation of the experiment design. **a** The process of apple seed isolation, germination, and hardening prior to introduction into microcosms; **b** Application of bacterial inoculants (*Serratia, Stenotrophomonas*, and their combination) and volatile compounds (2-nonanone, 2-butanone, 3-methyl-1-butanol, and volatile mixture) in environmental soil (ES1); as well as comparing the effect of the *Serratia* after application in environmental (ES2) and potting soil (PS). **c** Sampled compartments for microbial DNA extraction

The plant preparation involved seed extraction, germination, and seedling hardening, followed by introduction into microcosms and treatment application. Seeds were extracted from fresh fruits of the apple cultivar Gala and germinated on sterile water-soaked cotton (Fig. 1A). The Petri plates containing seeds were sealed with Parafilm, wrapped in aluminium foil, and stored at 4 °C for one month for cold stratification and germination. The seedlings were hardened by transferring them into 50 mL tubes containing water-soaked sterile cotton, and allowed to grow at room temperature and natural light for an additional week. After seven weeks, the seedlings were transplanted into soil in the prepared microcosms. The microcosms used in the current study are based on a design as previously described [29]. Briefly, they consist of two transparent plastic containers (0.5 L) which were fixed to each other (Fig. 1B). The upper chamber was fitted with an air filter (0.22 μm pore size) to prevent microorganisms from entering. The middle part of the device was fitted with a conical tube connecting the upper and lower chambers of the microcosm. The upper and lower chambers contained the plant phyllosphere and soil to which the treatments were applied (Fig. 1B). Treatments were applied in different soil types (i.e., environmental soil ES1 and ES2, as well as potting soil; PS). The environmental soil was characterized *as sandy loam with pH 7.55. The macronutrient levels were: phosphorus at 2113 mg/kg, potassium at 1848 mg/kg, magnesium at 6395 mg/kg, calcium at 12,860 mg/kg, and total sulfur at 522 mg/kg. The micronutrient levels comprised boron at 25.0 mg/kg, copper at 48.8 mg/kg, iron at 25,420 mg/kg, manganese at 718 mg/kg, zinc at 109 mg/kg, and sodium at 206 mg/kg.* The environmental soil was collected in two batches, separated by a timespan of seven months. The first batch of environmental soil (ES1) was used for application with volatiles and all microbial inoculants, while the second batch (ES2) involved the application of only *Serratia plymuthica* HRO C48 strain, and the effect compared between potting soil. *The potting soil was purchased from PATZER ERDEN GmbH (Sinntal-Altengronau, Germany), a commercial supplier of the classic substrate (CL-Pikier) for greenhouse farming, made from white peat and natural clay, with a pH of 5.8 and nutrient salts ranging from 1 to 2.5 g/l.* The potting soil and the two batches of environmental soil were sampled for microbiome analysis. All procedures including the addition of soil into the microcosms, seedling transplanting and treatment application were performed under sterile conditions. After seedling introduction and treatment application (n = 12) the assembled microcosms were positioned in a conditioned plant growth room with a defined temperature range (20–24 °C) and 16:8 h of light: darkness regimes for four weeks before sampling.

### Preparation and application of bacterial and volatile treatments

Bacterial cultures of *Serratia plymuthica* HRO C48 and *Stenotrophomonas rhizophila* SPA P69 were obtained from the microbial culture collection at the Institute of Environmental Biotechnology (Graz, Austria). Briefly, bacterial strains were streaked on nutrient agar, followed by inoculation of the bacterial colonies into 100 mL of Nutrient Broth (NB) II media (Merck, Darmstadt, Germany). The bacterial cultures were incubated in 300 mL flasks at 30 °C for 40 h on a shaker (115 rpm). Two flasks were prepared for each bacterial strain, and bacterial cultures with 10^7^ CFU were used for inoculation. The volatiles of the microbial inoculants were profiled using headspace solid-phase microextraction with gas chromatography mass spectrometry as described in the supplementary methods (*SM1*), together with the details of the emitted volatile profiles in Supplementary Data (SD1) and Figure S1. Three volatile compounds 2-nonanone, 2-butanone, and 3-methyl-1-butanol were selected based on their high prevalence in the headspace of *Serratia* and *Stenotrophomonas* bacterial cultures (Figure S1). Additionally, these compounds were shown in previous studies to play a significant role as messenger molecules in the mediation of plant-bacterial interactions [30]. Regarding 3-methyl-1-butanol, few studies have shown the production of this volatile compounds by microorganisms, albeit limited information regarding their potential role in microbe-microbe/plant interactions [31]. The treatment with the volatile mixture contained original product concentrations of the volatile compounds including 2-nonanone (0.5 µL of 0.82 g/mL); 2-butanone (20 µL of 0.805 g/mL) and 3-methyl-1-butanol (10 µL of 0.809 g/mL). The volatile compounds were added into 200 µL PCR tubes that were filled with cotton; and four tubes were buried into microcosms containing soil. All treatments including controls received 10 mL of sterile distilled water.

### Sample processing and DNA extraction

Four weeks after treatment application, soil, phyllosphere and roots samples were obtained. The phyllosphere samples comprised the aboveground plant parts (i.e., leaves and stem), while roots were sampled together with their attached soil (so-called the rhizosphere). Bulk soil samples were obtained from each microcosm. All samples were stored at − 70 °C. The phyllosphere and root were processed by grinding the material in a stomacher bag with 4 mL of 0.85% sodium chloride using a mortar and pestle. Thereafter, 2 mL of the homogenate was centrifuged at 16,000 g for 15 min at 4 °C. The supernatant was discarded and the pellet was frozen at -70 °C prior to DNA extraction using FastDNA™ SPIN Kit for Soil (MP Biomedicals; United States) following the manufacturer’s instructions. All the sample processing steps and DNA extraction were performed under a laminar flow hood.

### Amplicon library preparation and sequencing

The isolated DNA was used for amplicon library preparation involving the amplification of V3 and V4 regions of the bacterial 16S rRNA gene and ITS1/ITS2 regions of the fungal rRNA. Primer pairs including 515F/806R (515F: 5′-GTGCCAGCMGCCGCGGTAA-3′ and 806R: 5’-GGACTACHVGGGTWTCTAAT-3’) [32] and ITS1F/ITS2R (ITS1f: 5’-CTTGGTCATTTAGAGGAAGTAA-3’; ITS2r: 5’-GCTGCGTTCTTCATCGATGC-3’) [33] were used for library preparation of bacterial and fungal communities, respectively. The library preparation followed a one-step amplification using the primers pairs with sample-specific barcodes. For bacterial amplification, 1 µL of community DNA was used in each 30 µL reaction. The reaction mixture had 6 µL (5xTaq &GO, PCR pre-mix, MP Biomedicals), 0.6 µL (10 µM 515f/806r) primers, 0.45 µL of each peptide nucleic PCR clamps (50 µM mPNA and pPNA), and 20.9 µL of PCR grade water. The PNA PCR clamps were used to block the amplification of plastid and mitochondrial 16S rRNA gene sequences of plants during the PCR amplification of bacterial community DNA [34, 35]. All reactions were performed in triplicates on a thermocycler (Bio-Metra GmbH, Jena, Germany). The PCR program included: preheating (96 °C, ∞); initial denaturation (96 °C, 5 min); proceeded by 30 cycles of 94 °C denaturation, 60 s; 78 °C PNA step, 5 s; 54 °C annealing, 60 s; 74 °C elongation, 60 s; then 74 °C final elongation for 10 min.

The fungal community library preparation included the amplification of ITS1/ITS2 region in 30 µL reaction mixture; containing 6 µL (5xTaq &GO, PCR pre-mix, MP Biomedicals), 0.6 µL (10 µM ITS1f/ITS2r) primers, 21.8 µL (PCR grade water) and 1 µL DNA template. The cycler conditions involved a preheating step (95 °C, ∞), initial denaturation (96 °C, 300 s), 35 cycles of denaturation for 60 s at 96 °C, annealing for 60 s at 58 °C, and extension for 60 s at 74 °C, followed by final extension for 10 min at 74 °C. The amplicon size of the PCR products was checked by gel electrophoresis. Three technical replicates of each sample were pooled during the amplicon purification process, which was performed using Wizard® SV Gel and PCR Clean-Up System (Promega, Madison, WI), following the manufacturer’s instructions. The purified PCR amplicons were quantified using a NanoDrop™ spectrophotometer (Thermo Scientific, Wilmington, DE, USA). The samples were pooled in equal concentrations and sent for 250 paired-end Illumina MiSeq sequencing to MWG Eurofins (Ebersberg, Germany). The obtained raw data (i.e., fastq files for 16S rRNA gene fragments and the ITS region) are available in the European Nucleotide Archive (ENA) under accession number PRJEB61122.

### Quantification of bacterial and fungal abundance

Microbial abundance, estimated as gene copy numbers of bacteria and fungi in samples was performed using quantitative PCR using primer pairs Unibac-II-515f/Unibac-II-806r for bacteria (10 μM each; [36]) and ITS1f/ITS2r for fungi (10 μM each; [33]). Reactions were performed in a total volume of 10 μL in a reaction mix with 5 μL KAPA SYBR Green (Bio-Rad, Hercules, CA, U.S.A.), 0.5 μL of each primer, 3 μL PCR grade water and 1 μL template DNA. Samples were diluted 1:10 in PCR grade water. For each sample, amplifications were conducted in triplicates using the Rotor-Gene™ 6000 thermal cycler (Corbett Research, Sydney, Australia), with the following program: initial denaturation (95 °C, 5 min) followed by 35 cycles of denaturation (95 °C,10 s); annealing (54 °C, 15 s); extension (72 °C, 10 s); then melt down from 72 to 96 °C. Serial dilutions of standards containing defined copy numbers were used to calculate gene copy numbers. The bacterial and fungal community standards were prepared using DNA extracted from *Bacillus* sp., and *Penicillium* sp., respectively.

### Bioinformatics and statistical analysis

Raw amplicon sequencing datasets were denoised, joined, delineated into ASVs and assigned taxonomy in the QIIME 2 (v2023.9.1) environment (https://qiime2.org; [37]). First, paired-end raw reads were demultiplexed and primers removed using Cutadapt [38]. Demultiplexed sequence reads were quality checked using fastQC, followed by MultiQC-based summarization [39]. The datasets were then quality filtered, trimmed, denoised, merged, and chimeras removed using the DADA2 v1.26.0 pipeline [40], resulting into amplicon sequence variants (ASVs) and a table of feature counts. The representative sequences were subsequently taxonomically classified by alignment against the SILVA132 reference database [41, 42] and UNITE v7 [43] reference databases for the bacterial and fungal communities, respectively, using VSEARCH algorithm [44], implemented using the q2-feature-classifier command [45]. In addition, sequence datasets were used to generate a rooted phylogenetic tree using the plugin (qiime phylogeny align-to-tree-mafft-fasttree) with default parameters. The R version 4.0.3 [46] was used with phyloseq [47] and vegan [48] for statistical analysis and visualization.

The Shannon index and richness were calculated based on datasets that were rarefied to minimum sampling depths of 1387 and 2045 reads per sample for bacterial and fungal communities, respectively. Compartment-specific alpha rarefaction curves were generated using the ranacapa package [49]. The non-parametric Kruskal–Wallis and post-hoc Dunn’s tests with p-value correction by the Bonferroni method were used for the assessment of differences in microbial abundance and diversity among the different treatments, soil types, treatment type (i.e., bacteria or volatile treatment), and plant compartments. For community compositional analyses, the data were transformed using cumulative sum scaling (CSS) [50]. The Bray–Curtis dissimilarity was used to perform principal coordinate analysis (PCoA) and permutational analysis of variance (PERMANOVA, 999 permutations) to test community structure variations attributed to inoculant type (i.e., if the treatment was a volatile or bacterial inoculant), individual treatments, compartment, and soil type. The treatment categories were either bacterial and volatile inoculants or the non-treated control.

DESeq2 was used to test for differentially abundant taxa between treatments and control in the different compartments. Treatment-specific differentially abundant taxa (i.e., *p*-value = 0.05 and |log_2_(treatment/control) |> 1) in soil, root, and phyllosphere were used in the analysis of potential community functions that were affected by treatment application. Here, bacterial community functions were assigned based on taxonomy using the functional annotation of prokaryotic taxa (FAPROTAX) [51]. For fungal communities, FUNGuild was used to search for potential ecosystem functions associated with the fungal taxa [52].

### Carbon source utilization of the apple root microbiome after *Serratia* inoculation

Community-level ecosystem functions were assessed in the root for the experiment with the *Serratia* treatment. We used the Biolog EcoPlate system (Biolog Inc., CA, USA) to examine carbon source utilization patterns. Root samples were collected from microcosms, which were treated with *Serratia* for two soils (ES2 and PS). The samples were homogenized by grinding with 4 mL of 0.85% sterile sodium chloride, followed by centrifugation of the homogenized solution (500 g, 20 min) to obtain the supernatant containing the root microbiota. The supernatant was diluted by adding 6 mL (0.85% sodium chloride) to constitute 10 mL of the inoculum, followed by introduction of the supernatant into 96 well of the Biolog EcoPlate. For each sample, of which 100 μL was added into each well of the Biolog EcoPlate. Briefly, each plate contained with three technical replicates for water and 31 different carbon sources [53]. Two biological replicates were inoculated and the microplates were incubated in the dark at 20 °C. The absorbance of tetrazolium at 592 nm was measured at time intervals of approximately 12 h for 8 days using a SpectraMax microplate reader (ThermoFisher Scientific, Munich, Germany). For each sample, the time series were smoothed with a sliding window of five time points using linear regression (first order Savitzky-Golay filter) on the log-transformed absorbance values. From the first derivative of the smoothed absorbance values, we estimated exponential substrate utilization rates and extracted the maximal rates that were averaged for the two replicates.

## Results

### Bacterial volatiles and their effects on soil and plant microbiomes

When mixed, the bacterial cultures (*Serratia* and *Stenotrophomonas*) emitted more volatiles compared to each individual culture. The in vitro profiling of volatiles revealed that - twenty-five volatile compounds were produced by bacterial inoculants, where nine compounds were inoculant-specific and four were shared between single and mixed cultures (Figure S1).

Examining the effects of the two bacterial inoculants and their representative volatile compounds (i.e., 2-nonanone, 2-butanone, and 3-methyl-1-butanol) on plant and soil microbiomes involved analysis of amplicon sequencing datasets derived from samples obtained from the microcosms. Amplicon sequencing of the microbial community yielded 5,489,386 and 4,566,721 high-quality reads, respectively. After removing unassigned, mitochondrial, and chloroplast reads, we retained 4,275,237 and 4,566,721 reads, that could be assigned to 11,758 bacterial and 4,049 fungal ASVs. The selected sampling depths for bacteria (1387 reads/sample) and fungi (2045 reads/sample) sufficiently covered the phyllosphere microbial richness (Figure S2).

There was no significant difference in the Shannon diversity index between treatments and the control across the different compartments, except for the 2-butanone treatment which showed a significant increase in bacterial diversity in the phyllosphere (Figure S3). Bacterial richness (i.e., the number of observed ASVs) showed a similar pattern, albeit no significant differences (*P ≥ *0.05) between treatments and control were observed (Figure S3). Soil treated with *Serratia* showed a significantly higher fungal richness compared to the control (Figure S3). However, no treatments caused significant differences of fungal richness in the phyllosphere and root (Figure S3). Also, no significant effect on microbial evenness across the different compartments was observed (Figure S4A-B). The detailed statistical comparisons in microbial alpha diversity indices of different treatments in different compartments are shown in Table S1 and Table S2.

In terms of microbial abundance (estimated as 16S rRNA gene or ITS region copies), only the combination of volatiles significantly increased the phyllosphere bacterial abundance when compared to the control (*p = *0.05) (Figure S5, Table S3). There was also a significant increase (*p = *0.05) in the soil fungal abundance for the bacterial treatments (i.e., *Serratia, Stenotrophomonas*, and their combination) (Figure S5, Table S3); while no significant difference in fungal community abundance was observed in the root and phyllosphere for all treatments (Figure S5). Interestingly, these effects depended on the soil used. The *Serratia* treatment showed a significant reduction in soil bacterial abundance in potting soil, and a significant increase in soil fungal abundance in environmental soil.

### Treatment-induced microbial compositional shifts depend on compartment and inoculum type

PERMANOVA performed on Bray–Curtis dissimilarity matrices, revealed a significant effect of compartment (Bacterial: R^2^ = 32%, *p = *0.001; Fungal: R^2^ = 30%, *p = *0.001), treatment (Bacterial: R^2^ = 5%, *p = *0.001; Fungal: R^2^ = 7%, *p = *0.001) and treatment type (Bacterial: R^2^ = 2%, *p = *0.009; Fungal: R^2^ = 4%, *p = *0.001) on the microbial community structure. An interactive effect of compartment and treatment was found for microbial community composition (Bacterial: R^2^ = 7%, *p = *0.002; Fungal: R^2^ = 5%, *p = *0.02). The influence of different treatment types across plant compartment was also reflected in the PCoA clustering of Bray–Curtis dissimilarity matrices, for the bacterial and fungal communities, respectively Figure S6A-B. PERMANOVA was performed to compare the combined effect of treatment types (bacterial or volatile treatments) on the microbiome across plant compartments. Both treatment types significantly influenced the fungal community (i.e., Bacteria: R^2^ = 5%, *p = *0.003; volatiles: R^2^ = 3%, *p = *0.02), while the effect on the bacterial community was only observed for bacterial inoculants in the root compartment (Figure S6C). Comparison of inoculants relative to control showed that bacterial inoculants (i.e., *Serratia*, *Stenotrophomonas*, and consortium) significantly influenced the root bacterial communities, as well as the fungal communities across compartments (Figure S6). Volatile inoculants only showed a significant effect (*p = *0.05) on the soil and phyllosphere fungal community (Figure S6).

**Fig. 2:**
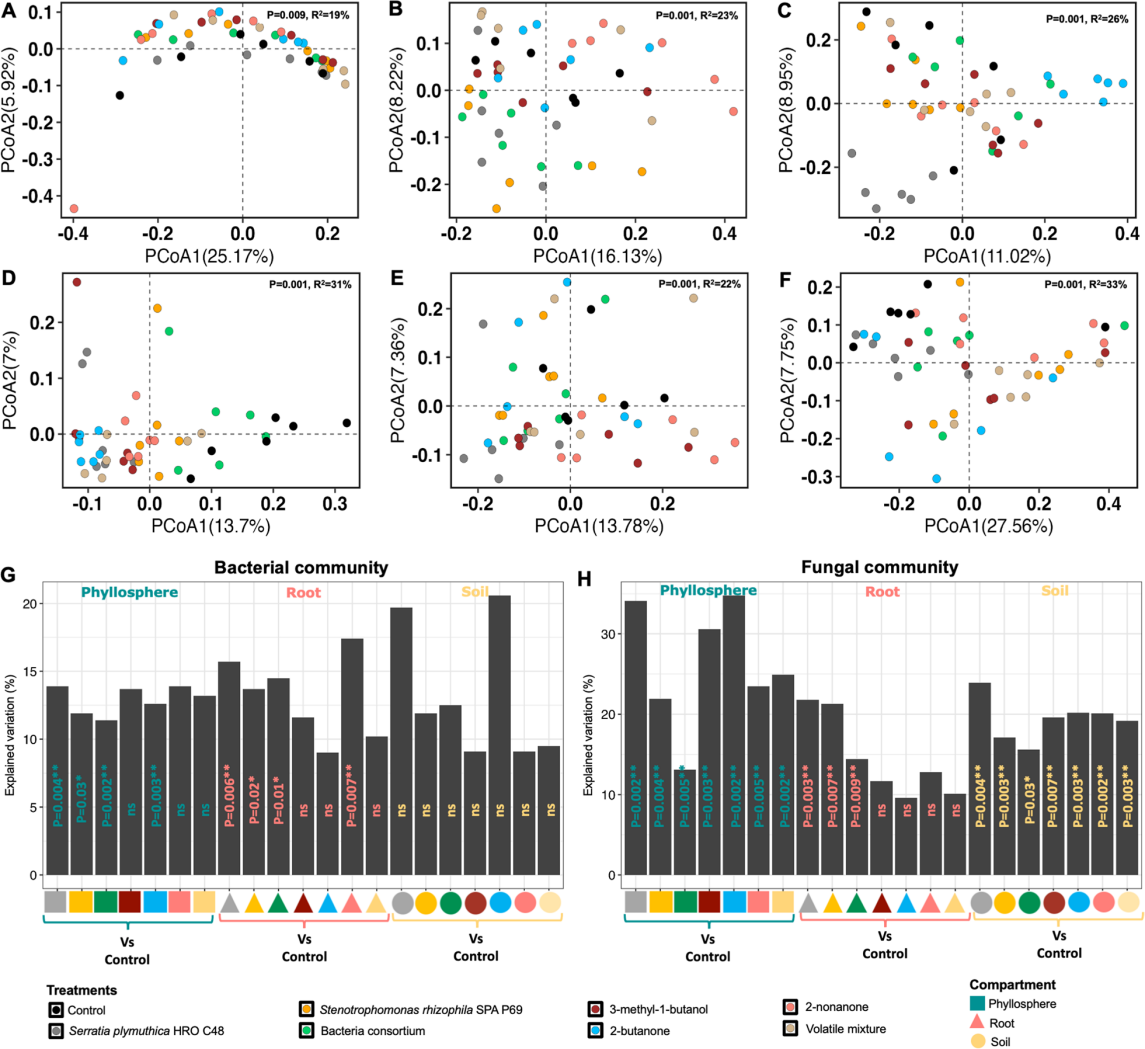
Treatments induced microbiome changes in a compartment-specific manner, especially in the phyllosphere and soil fungal communities. **A**–**C** are Principal Coordinates Analysis (PCoA) representations of Bray–Curtis dissimilarity matrices showing treatment effects on the bacterial community in the soil, root, and phyllosphere, **D**–**F** represent the same for fungal communities. Panels** G** and** H** show the percentage of variation explained by treatments based on pairwise PERMANOVA between treatments and the control. The colours and shapes represent treatments and plant compartments, respectively

Because there was a strong influence of the compartment on community composition, we examined the treatment effects in individual compartments such as the soil, root and phyllosphere. Significant compartment-specific effects of treatments on microbial communities were observed in: (i) soil (Bacterial: R^2^ = 19%, *p = *0.01; Fungal: R^2^ = 31%, *p = *0.001), (ii) root (R^2^ = 23%, *p = *0.001; R^2^ = 22%, *p = *0.001), and (iii) phyllosphere (R^2^ = 26%, *p = *0.001; R^2^ = 33%, *p = *0.001). Post-hoc analysis by pairwise PERMANOVA on individual treatments and the control was performed to examine the treatment effects in different compartments. There was a significant influence of all treatments on the soil and phyllosphere fungal community (Fig. 2H). Bacterial inoculants (i.e., *Serratia, Stenotrophomonas,* and consortium) had a significant effect on the bacterial community in the phyllosphere and root (Fig. 2G), as well as the fungal community in all compartments (Fig. 2H). The volatiles affected the fungal community in soil and the phyllosphere. A significant effect of volatile treatments on the root and phyllosphere bacterial community was only observed for 2-nonanone and 2-butanone treatments (Fig. 2G). The difference in microbiome structures between these treatments and the control was visualized by PCoA clustering of Bray–Curtis dissimilarity matrices for the bacterial (Fig. 2A–C) and fungal community (Fig. 2D–F), respectively. The bacterial inoculants exerted a stronger effect on the microbiome in the different compartments as compared to volatile inoculants. Moreover, among the bacterial treatments, *Serratia* caused the most significant shifts in the microbiome.

For significant pairwise comparisons between treatments and the control, and in different compartments (Fig. 2G and H), DESeq2 analysis was performed to uncover the differentially abundant ASVs that were collapsed to genus level, both for the bacterial and fungal community (Fig. S7A and S7B, respectively). Overall, bacterial inoculants contributed towards the enrichment of *Pseudomonas,* unidentified *Burkholderiaceae, Massillia, Methylobacillus, Methylotenera, Rhizobacter, Acidovorax, Xanthomonas, Flavobacterium* in the root, as well as the genera *Pelomonas, Cupriavidus, Methylobacterium,* and *Filimonas* in the phyllosphere (Figure S7A). The bacterial genus *Serratia* was significantly depleted in the root, both for treatments involving the volatile mixture and bacterial consortium; while *Erwinia* was depleted in the root in the *Stenotrophomonas* treatment (Figure S7A). Regarding the fungal community, bacterial inoculants contributed to the differential abundance of fungal genera in all compartments, while volatile treatments influenced the fungal taxa, only in soil and the phyllosphere. The genus *Penicillium* was enriched in the soil for volatile treatments, and was also enriched in the root for the treatment involving the bacterial combination (Figure S7B).

### The *Serratia* treatment induced microbiome shifts in a soil-specific manner

**Fig. 3:**
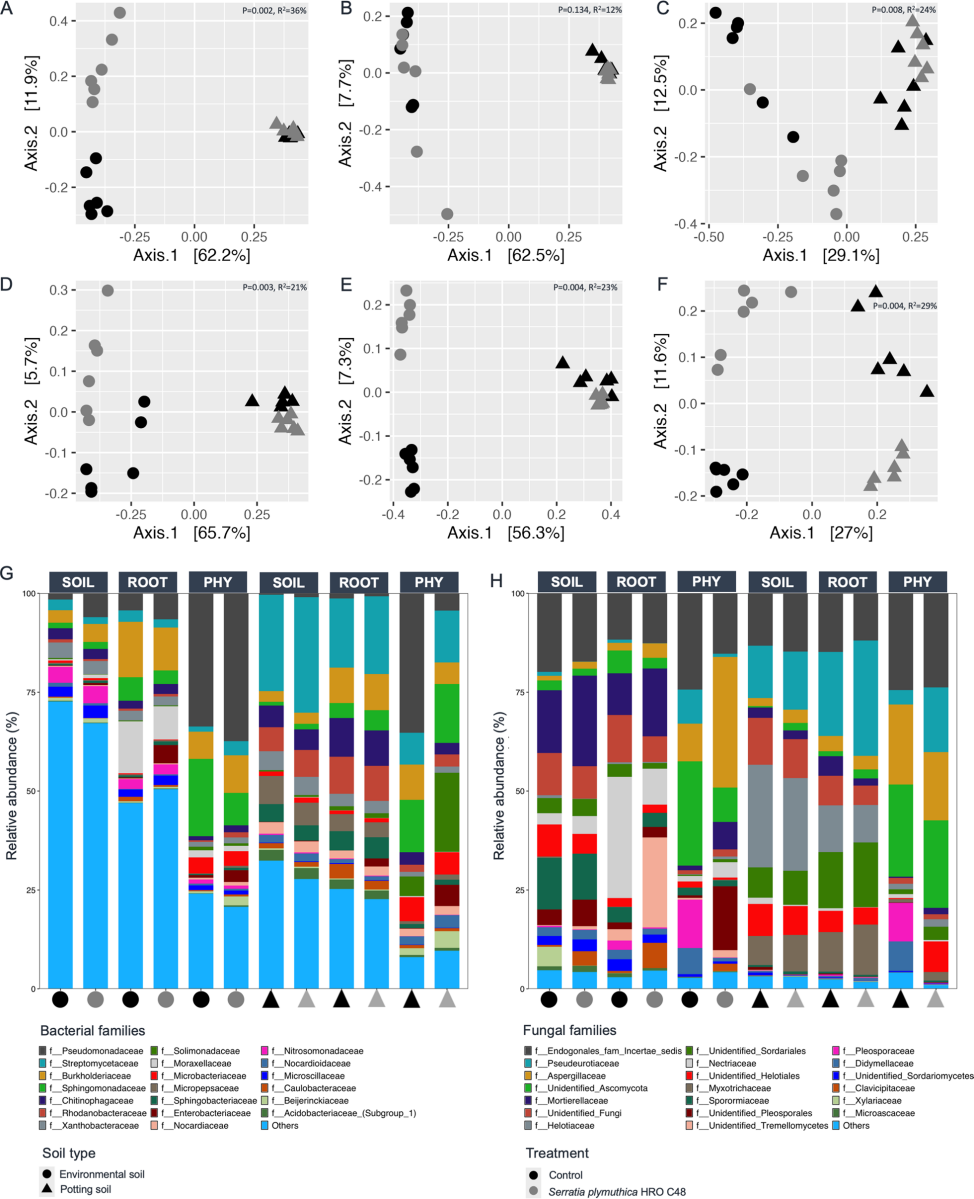
The effect of inoculation with *Serratia* on microbial community structures across soil type (i.e., environmental and potting soil, respectively). Panels **A**–**C** are principal coordinate analyses (PCoA) showing the bacterial community in different compartments for environmental (ES2: Circle), and potting soil (PS: Triangles). Panels **D**–**F** represent the same for the fungal community. Stacked bar-plots show percentage average relative abundance in microbial composition for the different compartments (soil, root, and phyllosphere: PHY) for samples (n = 6). The colours and shapes represent treatments and soil type, respectively

Owing to the observed effects of *Serratia* inducing microbiome shifts, a subsequent microcosm experiment was performed to examine the effects of *Serratia* under contrasting soil conditions involving environmental soil (ES2) and potting soil (PS) (Fig. 3). Because the environmental soils that were used in the first experiment (ES1) and the latter (ES2) were obtained from the same location at different time points we first examined by PERMANOVA on Bray–Curtis dissimilarity matrices the microbiome differences between all soils (i.e., ES1, ES2, and PS). Overall, there were differences in microbiome composition between the different soil types (Bacterial: R^2^ = 76%, *p = *0.001; Fungal: R^2^ = 64%, *p = *0.001) (Figure S8), indicating a batch effect in microbiome composition of the environmental soils obtained from the same location (Figure S8). The variation in soil types were also reflected in their taxonomic composition (Figure S8). Generally, there was significant effect of soil (Bacterial: R^2^ = 37%, *p = *0.001; Fungal: R^2^ = 38%, *p = *0.001), and *Serratia* treatment (Bacterial: R^2^ = 3%, *p = *0.004; Fungal: R^2^ = 3%, *p = *0.01), across plant compartments (Bacterial: R^2^ = 12%, *p = *0.001; Fungal: R^2^ = 8%, *p = *0.001). The differences in microbiome structure and taxonomic composition that were attributed to treatment and soil type were visualized by PCoA clustering and compartment-specific stacked barplots for the bacterial (Fig. 3A–C and G) and fungal communities (Fig. 3D–F and H), respectively. From the barplots, it is apparent from the proportional composition of taxa under the category “others” that the samples associated with environmental soil were more diverse in comparison to potting soil.

Except for the root bacterial community of environmental soil (Fig. 3B), the *Serratia* treatment was shown to significantly impact the bacterial and fungal communities in all compartments (Fig. 3A and D–F, Table S4). The *Serratia* treatment was also associated with a significant reduction (*p = *0.002) in the soil bacterial diversity and richness in environmental soil and associated with the significant reduction in soil and root bacterial diversity in potting soil (Figure S9). There was no significant effect (*P ≥ *0.05) of the treatment on the fungal diversity and richness in different compartments, both for environmental and potting soil, consistent with the previous experiment (Figure S9). There was a significant reduction in the soil bacterial abundance for potting soil treated with *Serratia* (Figure S5), as well as the significant increase in fungal abundance in samples obtained from the treated environmental soil. We deduce from above findings that inoculation with *Serratia* can induce shifts in the soil microbiome through increasing the soil microbial diversity.

### Microbial inoculants and volatile treatments contributed to enrichment of potential phyllosphere functions

**Fig. 4:**
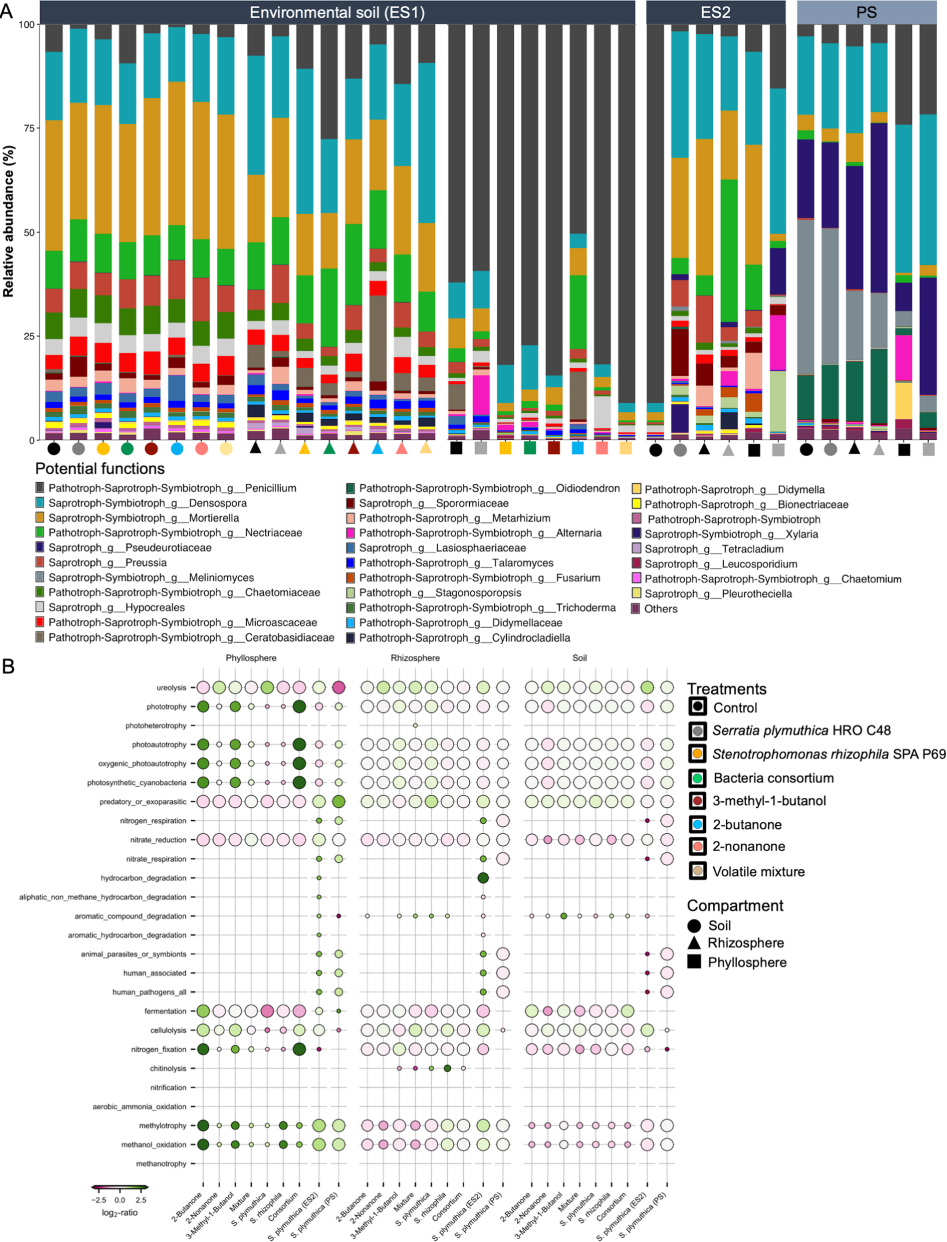
Taxonomy based prediction of putative microbiome functions using FUNGuild and FAPROTAX databases for the fungal and bacterial communities, respectively. Figure A shows the fungal community functional composition with the associated fungal genera for different compartments and treatments; applied in environmental soils (i.e., ES1 and ES2) and potting soil (PS). Treatments including volatile metabolites (i.e., 2-butanone, 2-nonanone, 3-methyl-1-butanol, and volatile mixture) and bacterial inoculants (i.e., *Serratia*, *Stenotrophomonas* and their combination) were applied in environmental soil (ES1); while *Serratia* was the only treatment that was applied in environmental soil (ES2) and potting soil (PS), respectively. The colours and shapes in panel** A** represent treatments and compartments, respectively. Panel **B** shows a dot plot representation of the bacterial community functions prediction by FAPROTAX for different compartments, treatments, and soil types. The dot size indicates functional abundance, and the colour scale indicates functional enrichment or depletion. The functional category assignment of the bacterial and fungal microbial community was performed using differentially abundant ASVs, as determined with DeSeq2 analyses

To examine the functions which were potentially affected by inoculants application, the taxonomy-based functional assignment tools FUNGuild [52] and FAPROTAX [51] were used for bacterial and fungal community functions, respectively. The FUNGuild database assigned functions to 191 ASVs which were differentially abundant in the different treatments relative to control; of these, only 131 ASVs could be allocated a function, while 60 ASVs were not assigned. The fungal community was majorly dominated by the presence of functional guild categories including saprotrophs, symbiotrophs, and pathotrophs (Fig. 4A). These functions were mainly driven by the fungal genera like *Penicillium, Densospora, Mortierella, Nectriaceae, Alternaria, Fusarium, Metarhizium,* and *Trichoderma*. Potential plant pathotrophs such as those categorized into the fungal genera *Fusarium, Alternaria*, and *Didymella* showed high prevalence in the root and phyllosphere.

Prediction of bacterial community functions by FAPROTAX revealed the presence of functions like nitrogen fixation, methylotrophy, methanol oxidation, phototrophy, and photoautotrophy especially in samples obtained from the phyllosphere (Fig. 4B). Moreover, all treatments were associated with the presence of functions like methylotrophy and methanol oxidation in the phyllosphere. Representative bacterial genera under the opportunistic human pathogen functional category included *Stenotrophomonas* spp. The taxonomic composition of all assigned functions in different plant compartments and treatments is detailed in Supplementary Data 2 and 3, for the bacterial and fungal functions, respectively.

### The *Serratia* treatment showed different carbon utilization in environmental and potting soil

**Fig. 5:**
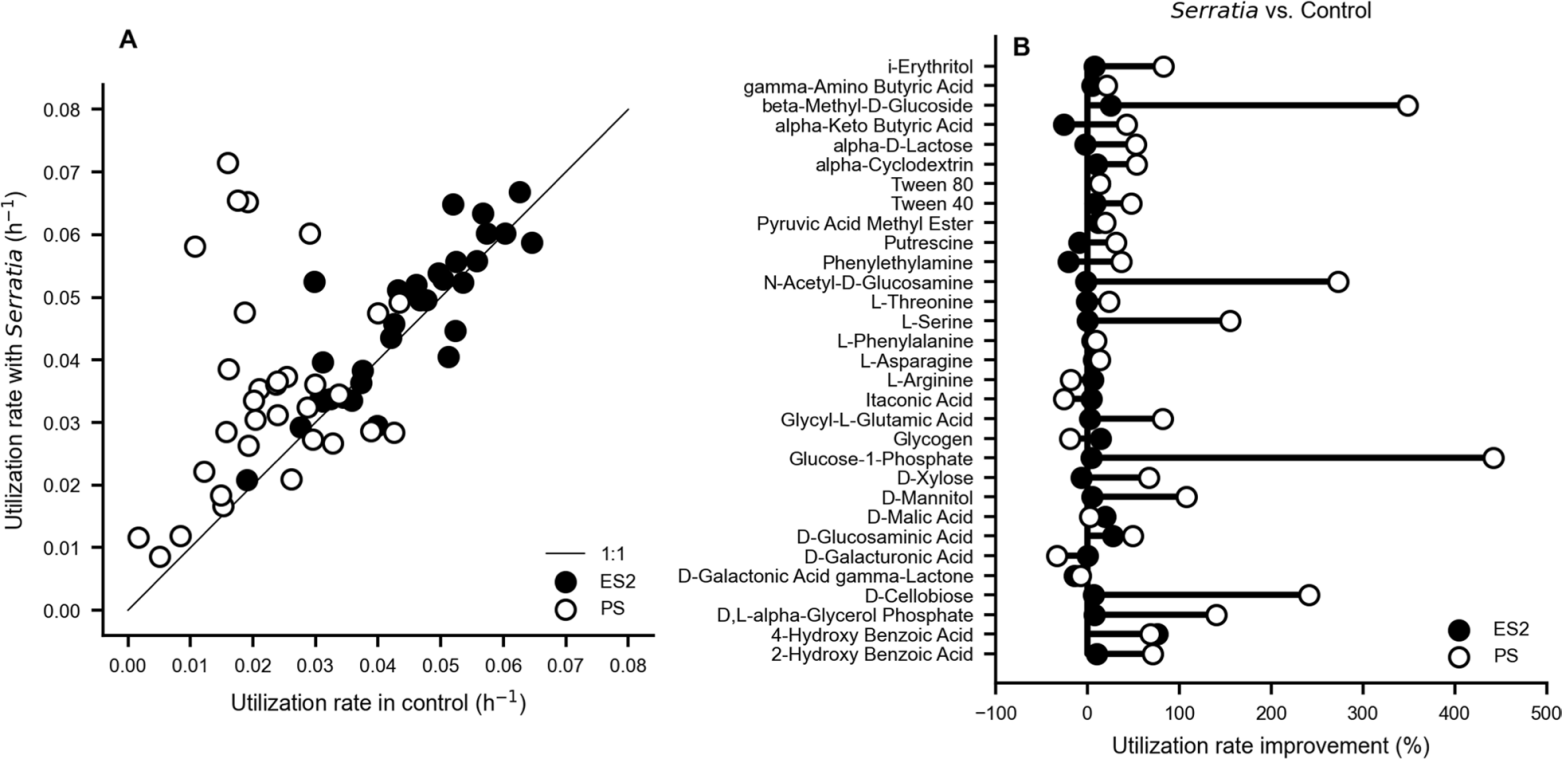
Carbon utilization patterns of the root microbiome after *Serratia* treatment. **A** Carbon utilization rates in the untreated control plotted against the rates obtained after treatment with *Serratia* for environmental soil (ES2) and potting soil (PS). **B** Utilization rate improvement for different carbon sources

Biolog EcoPlates were used to gain insights into shifts in carbon utilization due to the application of *Serratia* in environmental and potting soil. Overall, higher carbon utilization was observed for root samples associated with untreated environmental soil as compared to potting soil (Fig. 5A). The *Serratia* treatment as compared to control showed an increase in carbon substrate utilization in potting soil, while the utilization rates in environmental soil only increased by narrow margins for specific substrates such as 4-hydroxy benzoic acid, beta-methyl-D-glucoside, D-malic acid, and D-Glucosaminic acid (Fig. 5B). In potting soil, there was a large increase in substrate utilization due to *Serratia* treatment relative to the control. Here, high utilization of substrates including glucose-1-phosphate, beta-methyl-D-glucoside, L-serine, D-xylose, glycyl-L-glutamic acid, D, L-alpha-glycerol phosphate, D-cellobiose, N-acetyl-D-glucosamine, 4-hydroxy benzoic acid, and D-mannitol was observed (Fig. 5B). In contrast, the we observed a reduction in utilization of alpha-keto Butyric acid, Phenylethylamine, and D-Xylose, and D-Galactonic Acid gamma-Lactone in environmental soil that was treated with *Serratia*, as well as a reduction in the utilization rate of L-Arginine, Itaconic acid, Glycogen and D-Galacturonic acid. Altogether, adding bacterial inoculants to a complex soil microbiome such as environmental or potting soil can induce shifts in microbiome activity.

## Discussion

The present study showed that bacterial inoculants as compared to volatiles had a higher impact on plant and soil microbial community composition, and that the influence of bacterial inoculants was most apparent with the conducted *Serratia* treatment. The potential of *Serratia* and *Stenotrophomonas* based bacterial inoculants was shown in previous studies [10, 22]. For example, both bacteria emit bioactive volatiles which, in part, explains their potential to cause shifts in native soil and root microbiomes [54, 55], and their antagonistic activity against soil fungal pathogens [24, 26, 27, 54]. The present study indicates that all volatile and bacterial inoculants impacted the soil and phyllosphere fungal community. Bacterial inoculants had a stronger effect on the root and phyllosphere bacterial community than the impact that can be attributed to their volatiles. The two bacterial inoculants (i.e., *Serratia* and *Stenotrophomonas*), which were applied can be used as biofertilizers and bioprotectants in plant production. Both can influence the native soil and plant microbiomes (i.e., microbe-microbe interactions) through mechanisms like quorum sensing, biofilm formation, siderophore production, auxin biosynthesis, and through production of hydrolytic enzymes [26, 56, 57]. Therefore, a combination of traits underlying the bioactivity of these bacterial inoculants provides an explanation for the stronger effects in comparison the sole application of their volatiles. Moreover, it is important to highlight that volatile-mediated effects only partially account for microbe-microbe interactions, whereas the effects of their bacterial emitters are more diversified [58], but dependent on the ability to colonize and persist in the environment that they are applied.

The effects of volatiles on bacterial communities were observed in the phyllosphere and root, for 2-butanone and 2-nonanone treatments, respectively. Apart from the impact on the phyllosphere bacterial composition, 2-butanone was associated with a significant increase in the phyllosphere bacterial diversity. This observation was surprising because all treatments were applied in the belowground compartment of the microcosms. We assume that the volatile 2-butanone could have accumulated in the headspace of the microcosm, potentially supporting microorganisms that thrive on metabolizing volatile compounds [59, 60]. Moreover, microorganisms that are specialized inhabitants of the phyllosphere, such as methanotrophs can often degrade various low molecular weight compounds like volatiles compounds. The volatiles’ effects were mainly manifested on the soil fungal community composition. Soil contains a vast diversity of fungi, including various taxa that occur in filamentous forms [61]. The fungal community is likely more sensitive to volatiles owing to their hyphal network that spans across soil pores. This potentially explains the observed increased effect of volatiles on the soil fungal community composition.

The taxonomy-based predictions of microbiome functions showed prevalence of specific bacterial ecosystem functions like nitrogen fixation, methylotrophy, and methanol oxidation in the phyllosphere. These functions were also previously associated with the rice phyllosphere microbiome, and are crucial in the sustenance of the phyllosphere microbiome [59, 60, 62]. The *Serratia* treatment showed the presence of potential functions categorized as “human pathogen” in the phyllosphere, and this function was linked to *Stenotrophomonas* spp. While taxonomy-based functional predictions should be interpreted with caution, previous research has shown that various *Stenotrophomonas* spp. can be categorized as beneficial rhizobacteria [63]; however, *Stenotrophomonas maltophilia* can infect immunocompromised individuals as opportunistic human pathogen [64, 65]. The *Serratia* treatment was associated with an increase in the proportion of entomopathogenic fungal genera, which were represented by *Metarhizium* and *Trichoderma*, both in the root and soil. These genera are agriculturally important as bioprotectants [66, 67]. While taxonomy-based functional prediction tools such as FUNGuild can identify various functional guilds, a large proportion of the fungal community could not be assigned a function. This presents the need to expand coverage of this database, as earlier emphasized [52].

The microbial functional diversity can be deduced by examining the community level metabolic profiles. In this study, root samples that were obtained from *Serratia* treated soil (i.e., environmental or potting) showed a higher substrate utilization as compared to control; however, the utilization rate in potting soil was higher than for environmental soil, thus it is important to consider the native microbiota for microbiome management. The substrate utilization rate in root samples was consistent with prior findings [68–70]. The observed high utilization rate of the *Serratia* treatment with respect to the control can be associated to traits of *Serratia plymuthica* HRO C48 as a prolific root colonizer [57], with the potential to induce changes in soil or root microbial activity.

## Conclusions

This study demonstrates the impact of microbiome-targeted management strategies on native soil and plant microbiomes, using custom microcosms that separate the various plant compartments. Our experimental design was appropriate to show that the combined effect of bacterial inoculants was higher than combined effects of volatile treatments indicating that such compounds may only partially drive the microbiome-shaping activities of the utilized strains. Moreover, treatment effects on microbiome composition were compartment-specific and depend on the soil type. Taxonomy-based functional predictions were also found to be compartment- and soil-specific indicating varying effects across plant production systems. Assessment of the root microbiome’s metabolic activity showed that inoculation with *Serratia* enhanced the carbon utilization rate in different soils. Despite the implemented experimental design enabling the targeted assessment of the effects of volatiles, we could not delimit the potential effect that might be attributed to the escape of volatiles from the belowground compartment into the microcosm headspace.

## Supplementary Information

Supplementary file 1.

## Data Availability

The datasets generated during the current study are available in the European Nucleotide Archive (ENA) under the project number PRJEB61122, repository, [https://www.ebi.ac.uk/ena/browser/view/PRJEB61122]. All the codes for generating the figures in the manuscript can be accessed at https://github.com/kaboyo/Microbiome_Responses_to_Bioinoculants.
