# Supplementary material for: Plant microbiome responses to bioinoculants and volatiles
Source: Environ Microbiome. 2025 May 21;20:55. doi: 10.1186/s40793-025-00715-4 (PMC12096642; doi:10.1186/s40793-025-00715-4)
Supplement: Supplementary file 1 [file 40793_2025_715_MOESM1_ESM.docx]

**Plant microbiome responses to bioinoculants and volatiles**

Expedito Olimi^1,2^*, Martina Duller^1^, Martina Stangl^1^, Samuel Bickel^1^, Angelika Schaefer^1^, Peter Kusstatscher^1^, Wisnu Adi Wicaksono^1^, Ahmed Abdelfattah^3^, Tomislav Cernava^1,2^ and Gabriele Berg^1,3,4^*

***^1^****Institute of Environmental Biotechnology, Graz University of Technology, Graz, Austria*

***^2^****School of Biological Sciences, Faculty of Environmental and Life Sciences, University of Southampton, Southampton, United Kingdom*

***^3^****Leibniz Institute for Agricultural Engineering and Bioeconomy (ATB), Potsdam, Germany*

***^4^****Institute for Biochemistry and Biology, University of Potsdam, Potsdam, Germany*

*Corresponding author/s

Expedito Olimi, Gabriele Berg

Expedito Olimi: [expedito.olimi@tugraz.at](mailto:expedito.olimi@tugraz.at) (ORCID: 0000-0003-2549-0301)

Martina Duller: [martina.duller@student.tugraz.at](mailto:martina.duller@student.tugraz.at)

Martina Stangl: m.stangl@student.tugraz.at

Samuel Bickel: [samuel.bickel@tugraz.at](mailto:samuel.bickel@tugraz.at) (ORCID: 0000-0002-9839-4591)

Angelika Battisti: [angelika.battisti@tugraz.at](mailto:angelika.battisti@tugraz.at) (ORCID: 0009-0008-6711-1924)

Peter Kusstatscher: [peter.kusstatscher@tugraz.at](mailto:peter.kusstatscher@tugraz.at) (ORCID: 0000-0002-1168-7675)

Wisnu Adi Wicaksono: [wisnu.wicaksono@tugraz.at](mailto:wisnu.wicaksono@tugraz.at) (ORCID: 0000-0002-1556-1981)

Ahmed Abdelfattah: [AAbdelfattah@atb-potsdam.de](mailto:AAbdelfattah@atb-potsdam.de) (ORCID: 0000-0001-6090-7200)

Tomislav Cernava: [tomislav.cernava@tugraz.at](mailto:tomislav.cernava@tugraz.at) (ORCID: 0000-0001-7772-4080)

Gabriele Berg: [gabriele.berg@tugraz.at](mailto:gabriele.berg@tugraz.at) (ORCID: 0000–0001–9423–3101)

Supporting information

Supplementary methods (SM1): Volatile profiling of *Serratia plymuthica* HRO C48 and *Stenotrophomonas rhizophila* SPA P69 and the consortium

The bacterial genera Serratia and Stenotrophomonas are prolific producers of volatile compounds [1–3]. Thus, the volatile profiles of the two strains and their mixture were analysed. We used Headspace-Solid Phase Microextraction (HS-SPME) with gas chromatography-mass spectrometry (GC-MS) to measure the VOCs produced by the bacteria [4, 5] . Briefly, pure bacterial isolates were transferred with an inoculating loop onto approximately 10 mL nutrient agar (NA; Sifin, Berlin, Germany) in 20 mL headspace vials (75.5 × 22.5 mm; Chromtech, Idstein, Germany). Three replicates of individual bacterial isolates and the bacterial consortium were prepared for analysis. After 48 hours of incubation at room temperature, vials were sealed with crimp seals for VOCs analysis. The SPME was conducted with an automated sampler with a 50/30 µm Divinylbenzene/Carboxen/Polydimethylsiloxane (DVB/CAR/PDMS) StableFlex fiber with a length of 2 cm. Compounds present in the headspace were enriched for 30 minutes at 35 °C. The compound separation and detection, respectively, were performed using gas chromatography instrument (GC7890A, Agilent Technologies, Waldbronn, Germany), together with a quadruple mass analyser (MS5975C, Agilent Technologies, Waldbronn, Germany). Samples were run through a (5%-phenyl) methyl-polysiloxane column (i.e., 30m x 250 µm 0.25 µm: Length x Inner Diameter x Coating) (DB-5MS; Agilent Technologies, Waldbronn, Germany), followed by electron ionization (EI; 70 eV) and detection (mass range 25-350 AU). The inlet temperature was adjusted to 270 °C. For the temperature gradient the GC column was kept at 40 °C for 2 min, raised to 110 °C (rate of 5 °C/min), then again increased to 280 °C (rate 10 °C/min) and maintained at 280 °C for 3 min. The helium flow rate was set to 1.2 mL/min. The obtained spectra were compared with NIST MS Search 2.2 included in the software-package of the NIST 2014 [6]. The ratio of each peak was calculated as: the area of the peak divided through the total area of all peaks in the chromatogram, multiplied by 100%. Volatile compounds were identified based on their retention indices. Additionally, compound suggestion with the best relative spectrum match (RMatch) from the NIST14 database was manually checked. Together with the RI-Match, the identification suggestion was accepted or deleted. From this resulting peaks values data, the values of the respective controls (NA incubated with respective microorganism) were subtracted. The volatile profiles for the microbial isolates (Serratia, Stenotrophomonas, and consortium) were determined and represented in supplementary figure (S1), and the three volatile compounds (2-nonanone, 2-butanone, and 3-methyl-1-butanol) were selected for microbiome study.

Supplementary data (SD1). The microbial consortium emitted a higher number of volatile compounds compared to cultures of *Serratia* and *Stenotrophomonas*.

Generally, twenty five volatile compounds were emitted, and were found to be strain-specific; however, four compounds were shared between the bacterial strains and their consortium. Interestingly, 16 compounds were seen in the bacterial consortium, more than any single isolates (Figure S1). Three volatile compounds: 3-methyl-1-butanol (produced by all inoculants), 2-nonanone (produced only by *Serratia plymuthica* HRO C48), and 2-butanone (produced by *Stenotrophomonas rhizophila* SPA P69 and bacterial consortium) were selected for application into microcosms to study their effect on the plant and soil microbiome.


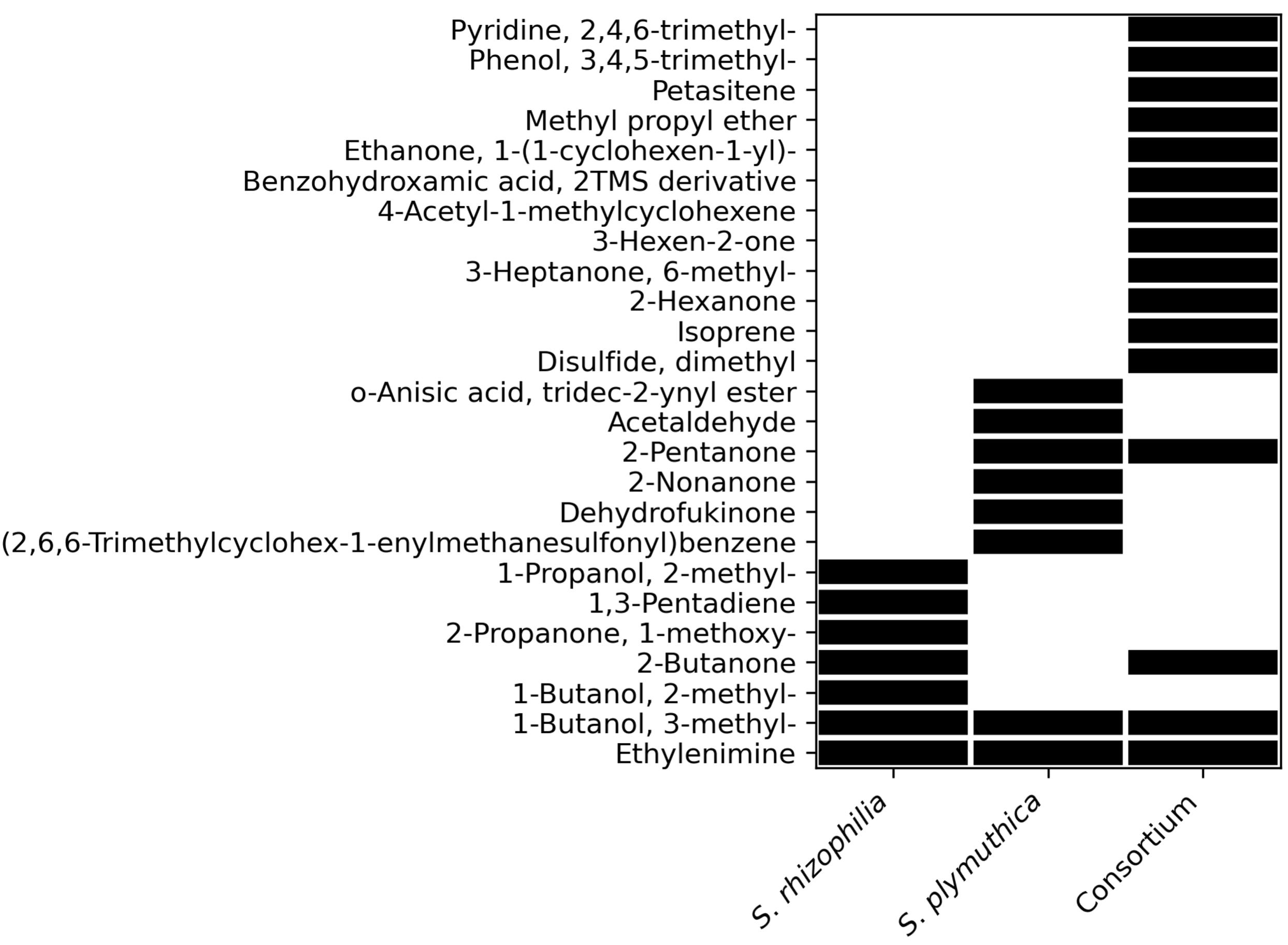


**Supplementary figure 1.** Occurrence of volatiles produced *in vitro* by *Serratia plymuthica* HRO C48, *Stenotrophomonas rhizophila* SPA P69 and their consortium.


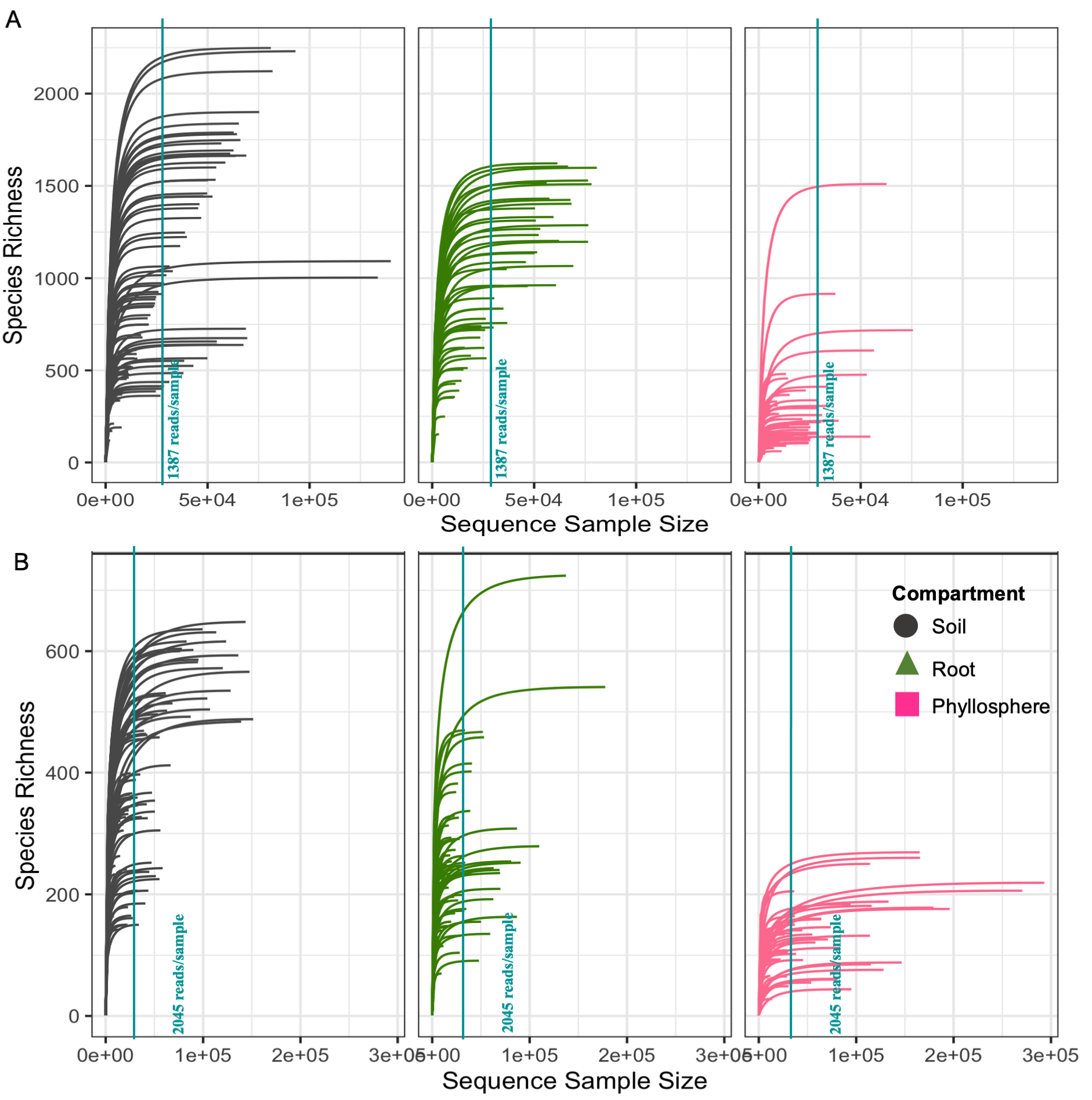


**Supplementary figure 2.** Rarefaction curves of bacterial (A) and fungal (B) richness in the soil, root and phyllosphere (black, green, and magenta, respectively). The vertical line indicates the minimum sampling depths (reads/sample) for bacterial (1387) and fungal (2045) communities.


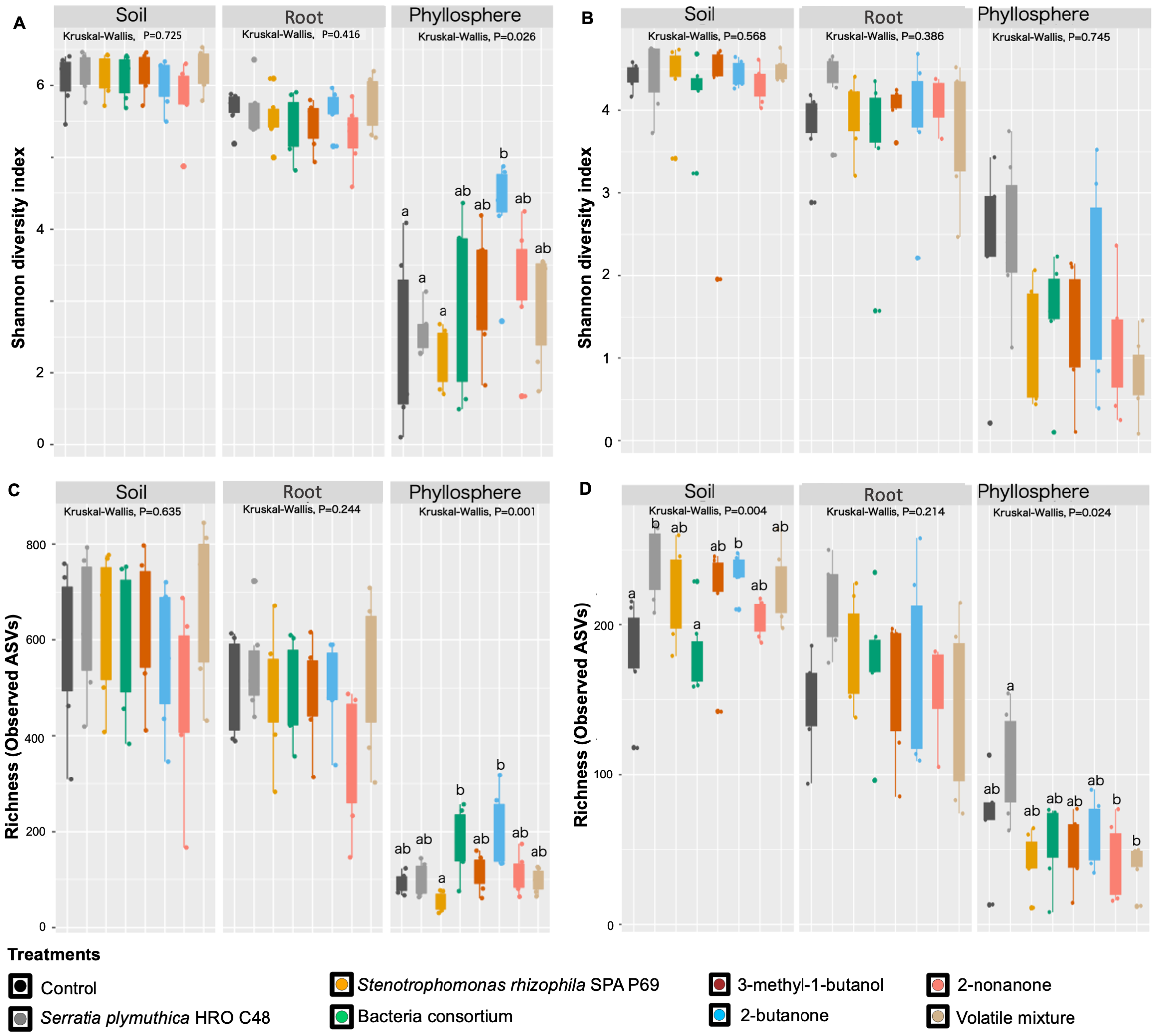


**Supplementary figure 3.** Boxplots showing microbial Shannon diversity and richness in different compartments for across treatments. Panels (A) and (B) show the bacterial and fungal Shannon diversity, while C and D show microbial richness. The boxplots represent sample distribution (n = 6) with 25^th^ and 75^th^ percentiles, while whiskers show maximum and minimum dataset. The letters indicated on the boxplots indicate significant differences between the different sample groups based on Dunn’s test (p ≤ 0.05). The data points outside the box area are outliers. The legend colours indicate the different treatments.


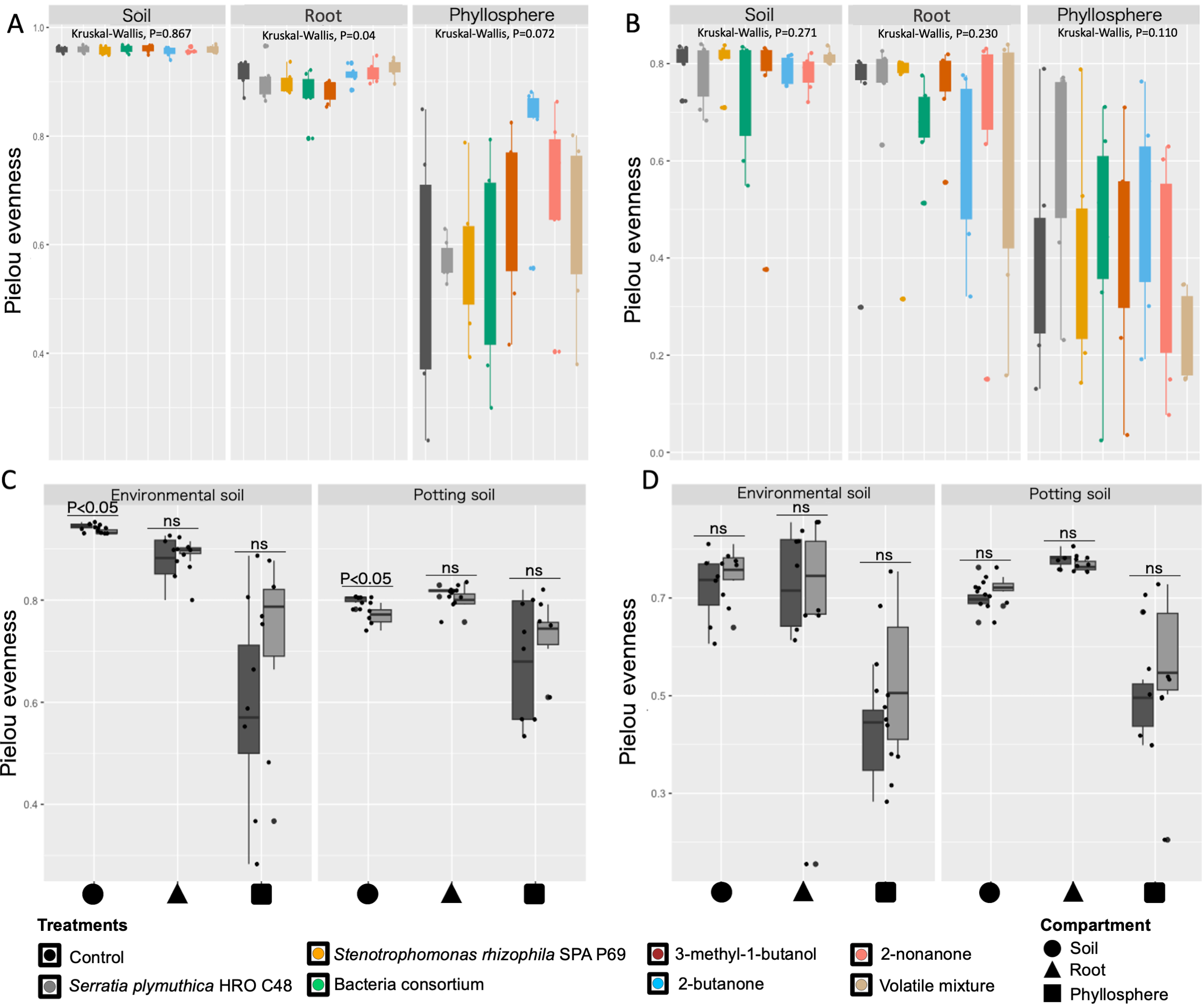
**Supplementary figure 4. Boxplot representation of the microbial evenness in the different compartments for the different treatments**. Panels (A) and (B) represent the compartment-specific bacterial and fungal evenness for different treatments applied in environmental soil. The letters on the boxplots represent significant differences between different sample groups (based on Dunn’s test). Panels (C) and (D) show the bacterial and fungal evenness comparisons between the Serratia treatment and control for experiment involving environmental-, and potting- soil, and samples taken for the different compartments. Pairwise Wilcox’s test was used to compare between Serratia and control. The boxplots represent sample distribution (n= 6 samples) including the 25^th^ and 75^th^ percentiles, while whiskers show maximum and minimum dataset. The data points outside the box area represent outliers. Legend colours represent treatments, while compartments type in panels (C) and (D) are shown as shapes. The boxplots lacking letters show treatments with no significant (ns) differences (p>0.05).


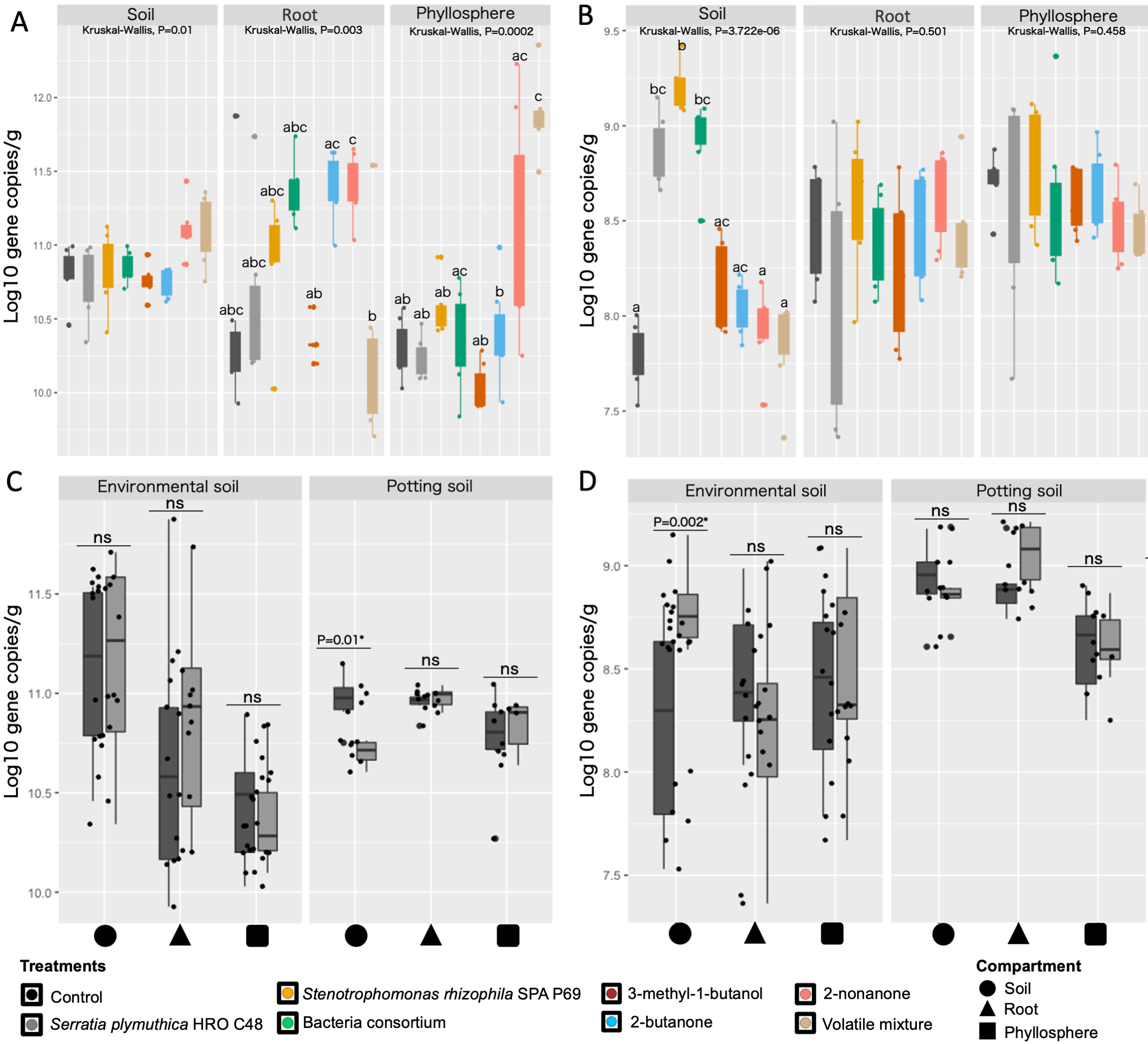


**Supplementary figure 5.** Boxplot representation of the microbial abundances in the different compartments for the bacterial and volatile treatments. Panels (A) and (B) represent the compartment-specific bacterial and fungal abundances for different treatments applied in environmental soil. The letters on the boxplots represent significant differences between the different sample groups (based on Dunn’s test). Meanwhile, panels (C) and (D) also represent bacterial and fungal abundance comparison between Serratia treatment and control which was performed in a follow-up experiment involving two soil types (environmental and potting soil), and samples taken from different compartments. Pairwise Wilcox’s test was used to compare between Serratia and control. The boxplots represent sample distribution (n=6 samples) with 25^th^ and 75^th^ percentiles, while whiskers show maximum and minimum dataset. The data points outside the box area represent outliers. The legend colours represent treatments, while compartments type in panels C and D are shown as shapes. The boxplots lacking letters show treatments with no significant (ns) differences (P>0.05).


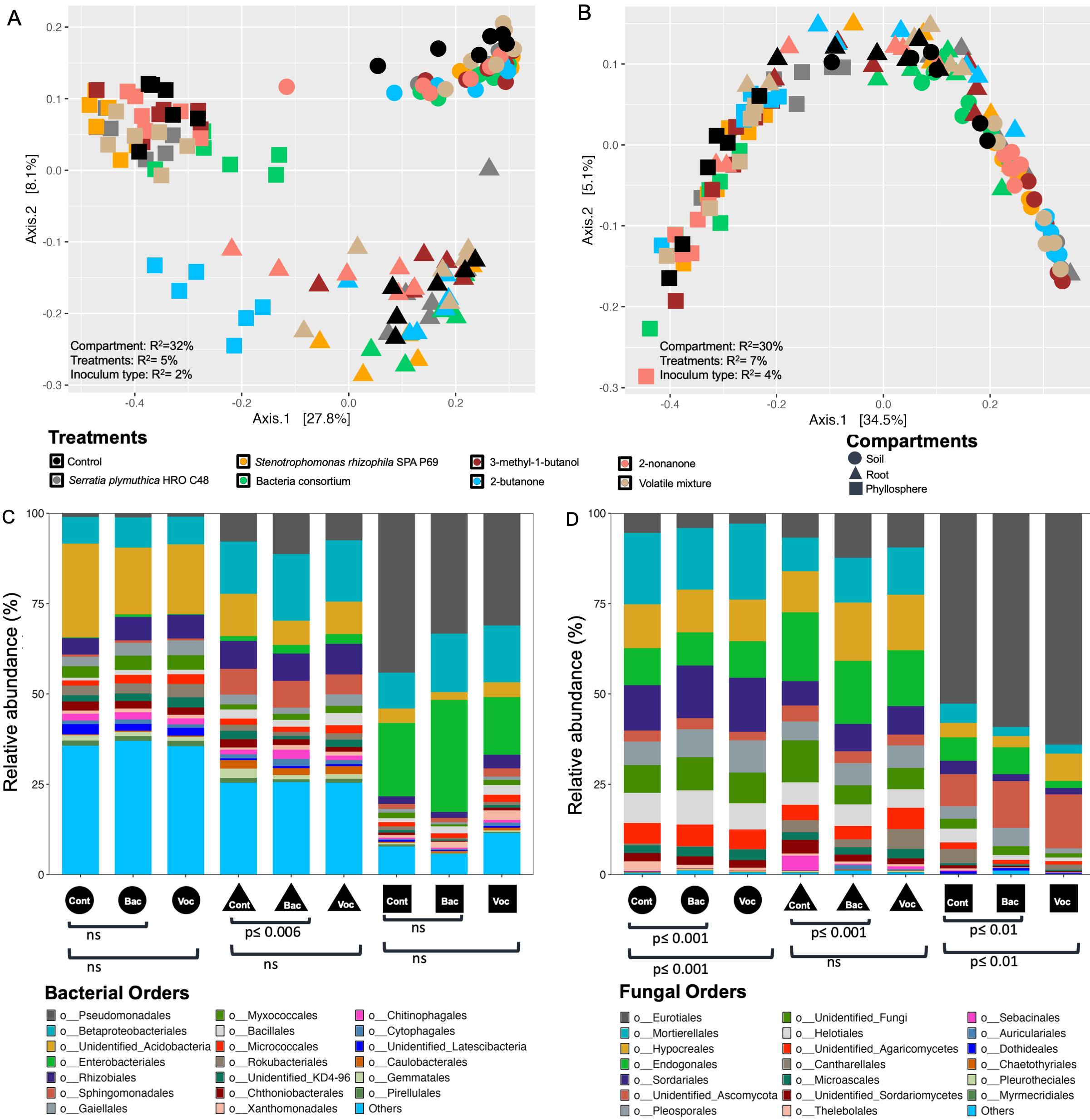


**Supplementary figure 6.** Panels A and B are Principal Coordinates Analysis (PCoA) showing the influence of treatment types across the plant compartments on the microbiome structure, both for bacterial and fungal community, respectively. Panels C and D are stacked barplots showing overall bacterial and fungal taxonomic composition at order level. Colours on figures A-B represent the different treatments while shapes represent compartments. Acronyms represent inoculant types including control (Cont), bacterial (Bac) and volatiles (Voc), respectively. The p-values are based on pairwise PERMANOVA between inoculant types and control.


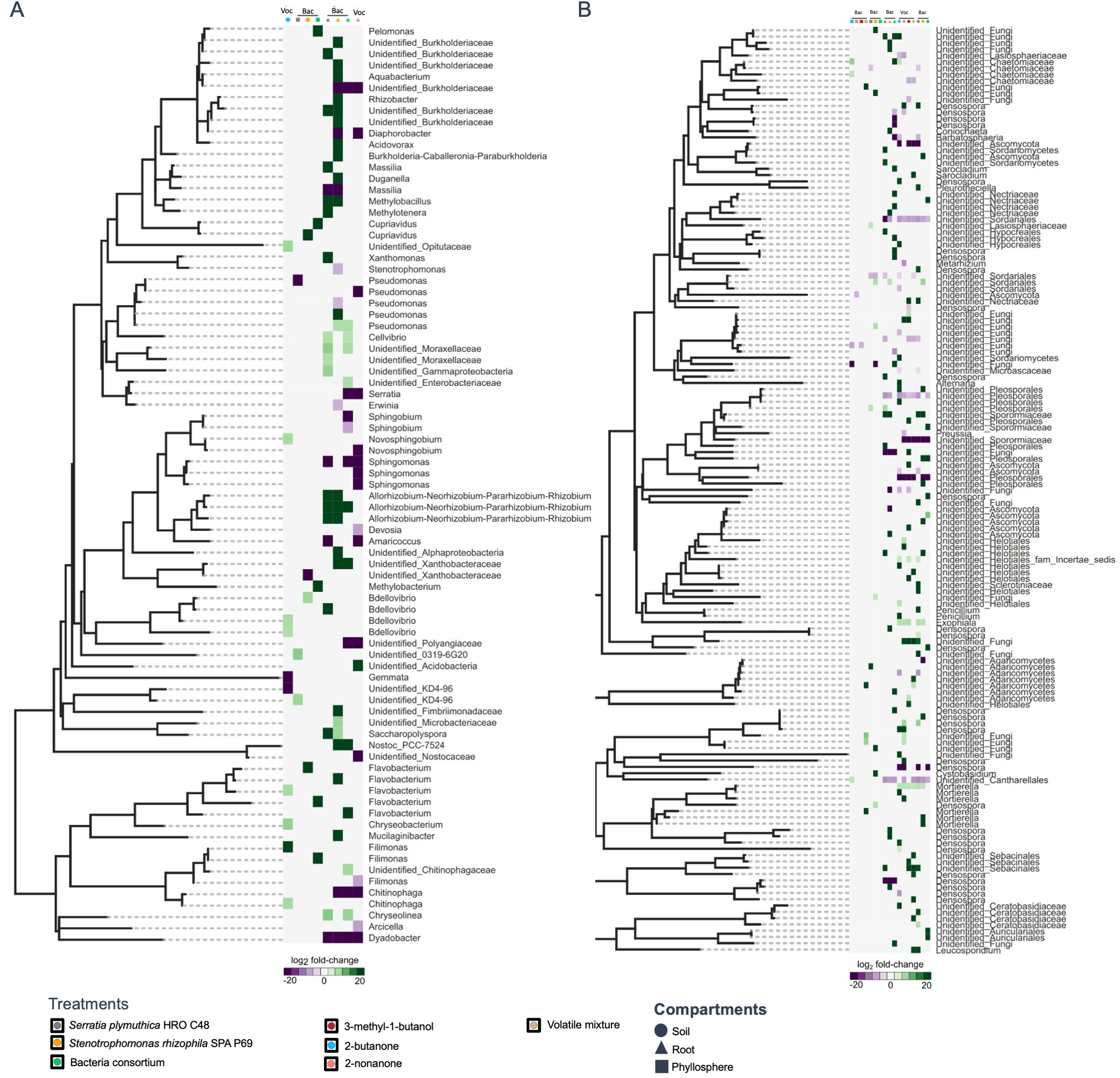


**Supplementary figure 7.** Phylogenetic trees representing the differentially abundant bacterial or fungal genera in the different compartment and treatment. The presented genera were selected basing on the log_2_ fold-change between -20 and 20, p≤0.05 by DESeq2 analysis. DESeq2 was performed only on treatments that were significantly different following pairwise PERMANOVA on treatments relative to control. Colours and shapes represent treatments and plant compartments, respectively. Treatments have been categorised into inoculum types as volatile (Voc) and Bacterial (Bac).

**
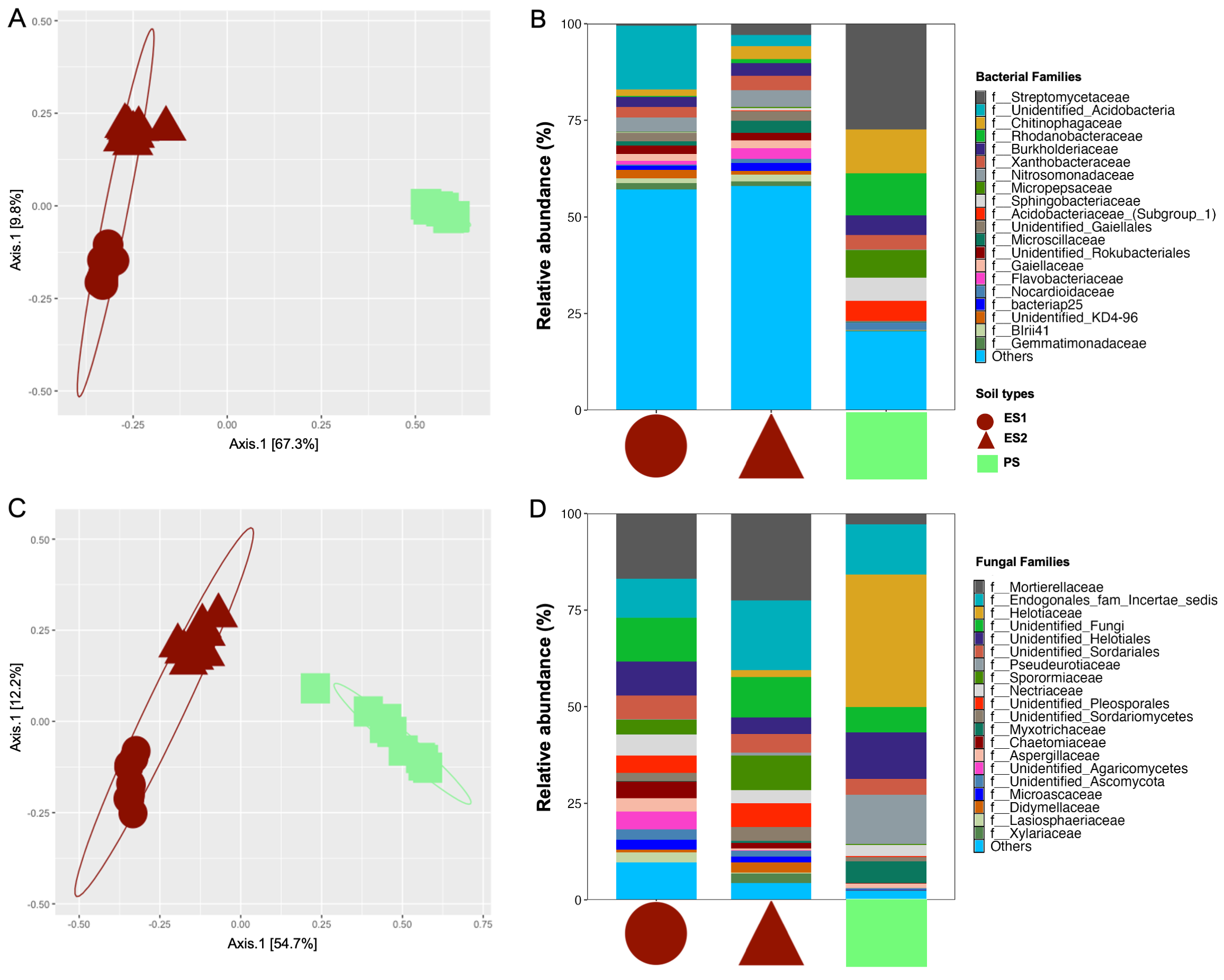
**

**Supplementary figure 8.** Microbial community composition between soil types which were used to study influence of microbiome management strategies with emphasis on Serratia plymuthica HRO C48. Panels (A) and (B) are Principal Coordinates Analysis plots and stacked bar plot representation of bacterial community composition in environmental soils (ES1 and ES2) and potting soil (PS); while (C) and (D) represent the same for the fungal community. Colours show different soil types, and shapes indicate the two environmental soils which were collected at two timepoints; each bar plot shows the average composition for n=10 samples.


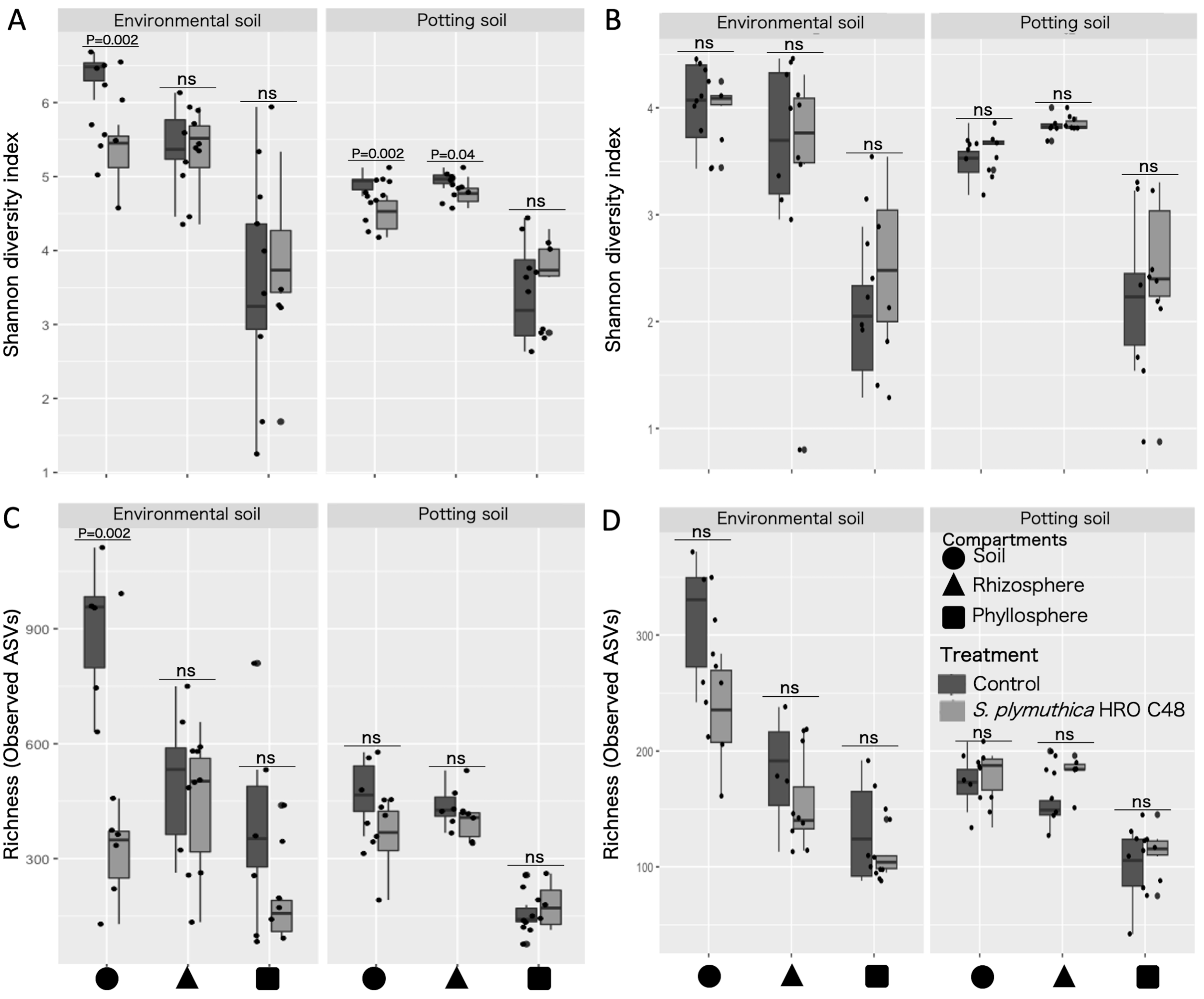


**Supplementary figure 9.** Boxplot representation of the microbial alpha diversity (i.e., richness and Shannon indices) comparison between Serratia treatment and control in different soil types and compartments. Panels (A) and (B) represent compartment-specific bacterial and fungal Shannon diversity index for Serratia and control in the two soils (environmental- and potting- soil). Meanwhile, (C) and (D) represent the same for microbial richness (observed ASVs). These results were obtained from follow-up experiment involving two soil types (environmental and potting soil) where Serratia was applied. The boxplots represent sample distribution (n=6 samples) with 25^th^ and 75^th^ percentiles, while whiskers show maximum and minimum dataset. Pairwise Wilcox’s test was used to compare between Serratia and control. The data points outside the box area represent outliers. The legend colours represent treatments, and compartments are shown by shapes. The acronym (ns) comparisons with no statistical significance (P>0.05). Shapes and colours represent compartment and treatment, respectively.

**Supplementary tables**

Supplementary table S1: Statistical comparison in microbial alpha-diversity (richness and Shannon diversity index) for the different treatments and compartments (i.e., non-parametric Kruskal-Wallis, KW’s).

| General effect of compartment and treatment on microbial richness (Observed ASVs) | | |
| --- | --- | --- |
|  | **Bacterial community** | **Fungal community** |
| General treatment effect (observed ASVs) | KW’s chi-squared = 3.30, df = 7, p = 0.855 | KW’s chi-squared = 8.373, df = 7, p = 0.301 |
| General compartment effect | KW’s chi-squared = 98.52, df = 2, p-value< 2.2e-16 | KW’s chi-squared = 98.94, df = 2, p-value < 2.2e-16 |
| Compartment-specific effects of treatment on microbial richness | | |
| Phyllosphere | **Bacterial community** | **Fungal community** |
| Overall effect of treatment in the phyllosphere | KW’s chi-squared = 25.07, df = 7, p = 0.001 | KW’s chi-squared = 16.12, df = 7, p = 0.024 |
| Pairwise treatment comparison | p. adjusted | p. adjusted |
| Control vs Serratia | ns | ns |
| Control vs Stenotrophomonas | ns | ns |
| Control vs Bacteria mixture | ns | ns |
| Control vs 3-methyl-1-butanol | ns | ns |
| Control vs 2-butanone | ns | ns |
| Control vs 2-nonanone | ns | ns |
| Control vs Volatile mixture | ns | ns |
| Root | **Bacterial community** | **Fungal community** |
| Overall effect of treatment in the Root | KW’s chi-squared = 7.046, Df = 7, P = 0.424 | KW’s chi-squared = 9.5728, df = 7, p = 0.214 |
| Pairwise treatment comparison | p. adjusted | p. adjusted |
| Control vs Serratia | ns | ns |
| Control vs Stenotrophomonas | ns | ns |
| Control vs Bacteria mixture | ns | ns |
| Control vs 3-methyl-1-butanol | ns | ns |
| Control vs 2-butanone | ns | ns |
| Control vs 2-nonanone | ns | ns |
| Control vs Volatile mixture | ns | ns |
| Soil | **Bacterial community** | **Fungal community** |
| Overall effect of treatment in the soil | KW’s chi-squared = 5.2014, df = 7, P = 0.635 | KW’s chi-squared = 20.758, df = 7, p = 0.004 |
| Pairwise treatment comparison | p. adjusted | p. adjusted |
| Control vs Serratia | ns | 0.027* |
| Control vs Stenotrophomonas | ns | ns |
| Control vs Bacteria mixture | ns | ns |
| Control vs 3-methyl-1-butanol | ns | ns |
| Control vs 2-butanone | ns | 0.038* |
| Control vs 2-nonanone | ns | ns |
| Control vs Volatile mixture | ns | ns |
| General effect of compartment and treatment on microbial Shannon diversity | | |
|  | **Bacterial community** | **Fungal community** |
| General treatment effect (Shannon) | KW’s chi-squared = 1.833, df = 7, p = 0.969 | KW’s chi-squared = 4.295, df = 7, p = 0.745 |
| General compartment effect | KW’s chi-squared = 111.94, df = 2, p-value< 2.2e-16 | KW’s chi-squared = 97.025, df = 2, p-value < 2.2e-16 |
| Compartment-specific effects of treatment on microbial Shannon diversity | | |
| Phyllosphere | **Bacterial community** | **Fungal community** |
| Overall effect of treatment in the phyllosphere | KW’s chi-squared = 15.94, df = 7, p = 0.026 | KW’s chi-squared = 13.01, df = 7, p = 0.072 |
| Pairwise treatment comparison | p. adjusted | p. adjusted |
| Control vs Serratia | ns | ns |
| Control vs Stenotrophomonas | ns | ns |
| Control vs Bacteria mixture | ns | ns |
| Control vs 3-methyl-1-butanol | ns | ns |
| Control vs 2-butanone | ns | ns |
| Control vs 2-nonanone | ns | ns |
| Control vs Volatile mixture | ns | ns |
| Root | **Bacterial community** | **Fungal community** |
| Overall effect of treatment in the Root | KW’s chi-squared = 7.12, df = 7, p = 0.416 | KW’s chi-squared = 7.418, df = 7, p = 0.386 |
| Pairwise treatment comparison | p. adjusted | p. adjusted |
| Control vs Serratia | ns | ns |
| Control vs Stenotrophomonas | ns | ns |
| Control vs Bacteria mixture | ns | ns |
| Control vs 3-methyl-1-butanol | ns | ns |
| Control vs 2-butanone | ns | ns |
| Control vs 2-nonanone | ns | ns |
| Control vs Volatile mixture | ns | ns |
| Soil | **Bacterial community** | **Fungal community** |
| Overall effect of treatment in the soil | KW’s chi-squared = 4.468, df = 7, p = 0.725 | KW’s chi-squared = 5.762, df = 7, p = 0.568 |
| Pairwise treatment comparison | p. adjusted | p. adjusted |
| Control vs Serratia | ns | ns |
| Control vs Stenotrophomonas | ns | ns |
| Control vs Bacteria mixture | ns | ns |
| Control vs 3-methyl-1-butanol | ns | ns |
| Control vs 2-butanone | ns | ns |
| Control vs 2-nonanone | ns | ns |
| Control vs Volatile mixture | ns | ns |

# Supplementary table S2: Statistical comparison in microbial evenness for the different treatments in the different compartments (i.e., non-parametric Kruskal-Wallis, KW’s).

| General Treatment effect on microbial (evenness) | Bacterial | Fungal community |
| --- | --- | --- |
| General treatment effect | KW’s chi-squared = 1.123, df = 7, p = 0.993 | KW’s chi-squared = 3.8989, df = 7, p = 0.791 |
| General compartment effect | KW’s chi-squared = 123.18, df = 2, p-value< 2.2e-16 | KW’s chi-squared = 89.546, df = 2, p-value < 2.2e-16 |
| Compartment-specific effects of treatment on microbial evenness | | |
| Phyllosphere | **Bacterial community** | **Fungal community** |
| Overall effect of treatment in the phyllosphere | KW’s chi-squared = 12.995, df = 7, p = 0.072 | KW’s chi-squared = 11.715, df = 7, p = 0.110 |
| Comparison comparisons | p. adjusted | p. adjusted |
| Control vs Serratia | ns | ns |
| Control vs Stenotrophomonas | ns | ns |
| Control vs Bacteria mixture | ns | ns |
| Control vs 3-methyl-1-butanol | ns | ns |
| Control vs 2-butanone | ns | ns |
| Control vs 2-nonanone | ns | ns |
| Control vs Volatile mixture | ns | ns |
| Root | **Bacterial community** | **Fungal** |
| Overall effect of treatment in the Root | KW’s chi-squared = 14.427, df = 7, P = 0.044 | KW’s chi-squared = 9.3377, df = 7, p = 0.230 |
| Comparison comparisons | p. adjusted | p. adjusted |
| Control vs Serratia | ns | ns |
| Control vs Stenotrophomonas | ns | ns |
| Control vs Bacteria mixture | ns | ns |
| Control vs 3-methyl-1-butanol | ns | ns |
| Control vs 2-butanone | ns | ns |
| Control vs 2-nonanone | ns | ns |
| Control vs Volatile mixture | ns | ns |
| Soil | **Bacterial community** | **Fungal community** |
| Overall effect of treatment in the soil | KW’s chi-squared = 3.191, df = 7, P = 0.867 | KW’s chi-squared = 8.7534, df = 7, p = 0.271 |
| Comparison comparisons | p. adjusted | p. adjusted |
| Control vs Serratia | ns | ns |
| Control vs Stenotrophomonas | ns | ns |
| Control vs Bacteria mixture | ns | ns |
| Control vs 3-methyl-1-butanol | ns | ns |
| Control vs 2-butanone | ns | ns |
| Control vs 2-nonanone | ns | ns |
| Control vs Volatile mixture | ns | ns |

# Supplementary table S3: Statistical comparisons of microbial abundance for the different treatments in the different compartments.

| General effect of compartment and treatment on microbial abundance | | |
| --- | --- | --- |
|  | **Bacterial community** | **Fungal community** |
| General treatment effect | KW’s chi-squared = 34.17, df = 7, P = 1.599e-05 | KW’s chi-squared = 25.36, Df = 7, P = 0.001 |
| Compartment-specific effects of treatment on microbial abundance | | |
| Phyllosphere | **Bacterial community** | **Fungal community** |
| Overall effect of treatment in the phyllosphere | KW’s chi-squared = 28.52, df = 7, P = 0.0002 | KW’s chi-squared = 6.726, Df = 7, P = 0.458 |
| Pairwise treatment comparison | p. adjusted | p. adjusted |
| Control vs Serratia | ns | ns |
| Control vs Stenotrophomonas | ns | ns |
| Control vs Bacteria mixture | ns | ns |
| Control vs 3-methyl-1-butanol | ns | ns |
| Control vs 2-butanone | ns | ns |
| Control vs 2-nonanone | ns | ns |
| Control vs Volatile mixture | 0.007* | ns |
| Root | **Bacterial community** | **Fungal community** |
| Overall effect of treatment in the Root | KW’s chi-squared = 22.011, df = 7, P = 0.003 | KW’s chi-squared = 6.3365, Df = 7, P = 0.501 |
| Pairwise treatment comparison | p. adjusted | p. adjusted |
| Control vs Serratia | ns | ns |
| Control vs Stenotrophomonas | ns | ns |
| Control vs Bacteria mixture | ns | ns |
| Control vs 3-methyl-1-butanol | ns | ns |
| Control vs 2-butanone | ns | ns |
| Control vs 2-nonanone | ns | ns |
| Control vs Volatile mixture | ns | ns |
| Soil | **Bacterial community** | **Fungal community** |
| Overall effect of treatment in the soil | KW’s chi-squared = 18.322, df = 7, P = 0.011 | KW’s chi-squared = 37.53, Df = 7, P = 3.722e-06 |
| Pairwise treatment comparison | p. adjusted | p. adjusted |
| Control vs Serratia | ns | 0.003* |
| Control vs Stenotrophomonas | ns | 0.000* |
| Control vs Bacteria mixture | ns | 0.003* |
| Control vs 3-methyl-1-butanol | ns | ns |
| Control vs 2-butanone | ns | ns |
| Control vs 2-nonanone | ns | ns |
| Control vs Volatile mixture | ns | ns |

# Supplementary table S4: A follow-up experiment testing the effect of Serratia plymuthica HRO-C64 on the microbial community composition (bacterial:16S rDNA; fungal: ITS) in different compartments, for different soils. Asterix represent significant p values such that: p≤0.001 (***), p≤0.01 (**) and p≤0.05 (*).

|  | Bacterial community | | | | Fungal community | | |
| --- | --- | --- | --- | --- | --- | --- | --- |
|  | df | R2 (%) | P (>F) |  | R2 (%) | P (>F) |  |
| Plant compartment | 2 | 12 | 0.001 | *** | 8 | 0.001 | *** |
| Treatment | 1 | 3 | 0.004 | ** | 3 | 0.001 | *** |
| Soil type | 1 | 37 | 0.001 | *** | 38 | 0.001 | *** |
| Plant compartment * treatment | 2 | 2 | 0.05 | . | 3 | 0.03 | * |
| Plant compartment * soil type | 2 | 8 | 0.001 | *** | 4 | 0.001 | *** |
| Treatment*soil type | 1 | 2 | 0.01 | * | 3 | 0.001 | *** |
| Plant compartment*treatment* soil type | 2 | 2 | 0.02 | * | 3 | 0.02 | * |
| Residual | 60 | 34 |  |  | 35 |  |  |
| Total | 71 | 100 |  |  | 100 |  |  |
|  | **Environmental soil** | | | | | | |
|  | **Bacterial community** | | | | **Fungal community** | | |
|  | df | R2 (%) | P (>F) |  | R2 (%) | P (>F) |  |
| Plant compartment | 2 | 23 | 0.001 | *** | 21 | 0.001 | *** |
| Treatment | 1 | 10 | 0.001 | *** | 13 | 0.001 | *** |
| Plant compartment * treatment | 2 | 8 | 0.006 | ** | 7 | 0.006 | ** |
| Residual | 30 | 60 |  |  | 59 |  |  |
| Total | 35 | 100 |  |  | 100 |  |  |
|  | **Phyllosphere (Environmental soil)** | | | | | | |
| Treatment | 1 | 24 | 0.01 | ** | 29 | 0.004 | ** |
| Residual | 10 | 76 |  |  | 71 |  |  |
| Total | 11 | 100 |  |  | 100 |  |  |
|  | **Root (Environmental soil)** | | | | | | |
| Treatment | 1 | 11.6 | 0.02 | . | 23 | 0.004 | ** |
| Residual | 10 | 80.4 |  |  | 77 |  |  |
| Total | 11 | 100 |  |  | 100 |  |  |
|  | **Soil (Environmental soil)** | | | | | | |
| Treatment | 1 | 36 | 0.002 | ** | 21 | 0.006 | ** |
| Residual | 14 | 63 |  |  | 79 |  |  |
| Total | 16 | 100 |  |  | 100 |  |  |
|  | **Potting soil** | | | | | | |
|  | **Bacterial community** | | | | **Fungal community** | | |
|  | df | R2 (%) | P (>F) |  | R^2^ (%) | P (>F) |  |
| Plant compartment | 2 | 47 | 0.001 | *** | 26 | 0.001 | *** |
| Treatment | 1 | 3 | 0.05 | . | 9 | 0.001 | *** |
| Plant compartment * treatment | 2 | 5 | 0.11 |  | 11 | 0.001 | *** |
| Residual | 30 | 45 |  |  | 55 |  |  |
| Total | 35 | 100 |  |  | 100 |  |  |
|  | **Phyllosphere (Potting soil)** | | | | | | |
| Treatment | 1 | 13 | 0.02 | * | 27 | 0.003 | ** |
| Residual | 10 | 87 |  |  | 73 |  |  |
| Total | 11 | 100 |  |  | 100 |  |  |
|  | **Root (Potting soil)** | | | | | | |
| Treatment | 1 | 12 | 0.13 |  | 26 | 0.004 | ** |
| Residual | 10 | 88 |  |  | 74 |  |  |
| Total | 11 | 100 |  |  | 100 |  |  |
|  | **Soil (Potting soil)** | | | | | | |
| Treatment | 1 | 23 | 0.02 | * | 22 | 0.001 | ** |
| Residual | 10 | 77 |  |  | 78 |  |  |
| Total | 11 | 100 |  |  | 100 |  |  |

References

1. Kai M, Effmert U, Berg G, Piechulla B. Volatiles of bacterial antagonists inhibit mycelial growth of the plant pathogen Rhizoctonia solani. Archives of Microbiology. 2007;187:351–60.

2. Kai M, Crespo E, Cristescu SM, Harren FJM, Francke W, Piechulla B. Serratia odorifera: Analysis of volatile emission and biological impact of volatile compounds on Arabidopsis thaliana. Applied Microbiology and Biotechnology. 2010;88:965–76.

3. Kai M, Haustein M, Molina F, Petri A, Scholz B, Piechulla B. Bacterial volatiles and their action potential. Applied Microbiology and Biotechnology. 2009;81:1001–12.

4. Verginer M, Leitner E, Berg G. Production of volatile metabolites by grape-associated microorganisms. Journal of Agricultural and Food Chemistry. 2010;58:8344–50.

5. Cernava T, Aschenbrenner IA, Grube M, Liebminger S, Berg G. A novel assay for the detection of bioactive volatiles evaluated by screening of lichen-associated bacteria. Frontiers in Microbiology. 2015;6 MAY:398.

6. MLA. NIST chemistry WebBook. Choice Reviews Online. 1997;35:35SUP-215-35SUP – 215.
